# Health Care–Related Determinants of First-Time Long-Term Care Need in Older Adults in Germany: Retrospective Cohort Study Using Claims Data

**DOI:** 10.2196/86572

**Published:** 2026-07-20

**Authors:** Till Baldenius, Kathrin Jürchott, Christine Haeger, Susann Behrendt, Stefan Blüher, Susanne Schnitzer

**Affiliations:** 1 AOK Research Institute (WIdO), Rosenthaler Straße 31, Berlin, 10178, Germany, 49 3034646 ext 3619; 2 Institute of Medical Sociology and Rehabilitation Science, Charité – University Medical Center Berlin, Corporate Member of Freie Universität Berlin and Humboldt-Universität zu Berlin, Berlin, Germany

**Keywords:** patient care, health services for the aged, long-term care, preventive health services, cohort studies, retrospective studies, routinely collected health data, aged, early detection of cancer, vaccination, specialization, ambulatory care, physical therapy modalities, drug prescriptions, Germany, epidemiology

## Abstract

**Background:**

In recent years, German long-term care (LTC) insurance has experienced an unprecedented increase in the number of beneficiaries. This raises the question of the role of health care in preventing or delaying the need for LTC. At present, related findings are limited. Addressing this research gap could promote longevity and improve quality of life while reducing the financial strain on the social security system.

**Objective:**

This study aimed to investigate the associations between the utilization of health care services and first-time LTC need.

**Methods:**

This retrospective cohort study examined nationwide linked claims data from the German statutory health and LTC insurance fund, AOK. The dataset included all individuals aged ≥60 years. Using multiple logistic regression, we investigated the association between health care utilization during the 5-year exposure period from 2016 to 2020 and the occurrence of first-time LTC need during the first quarter of 2021. Physician care, pharmaceutical care, physiotherapy, and medical aids were analyzed while adjusting for age, sex, regional variables, and comorbidities. Metrically scaled variables were categorized using the Fisher-Jenks algorithm to explore possible nonlinearities. Individuals who needed LTC prior to 2021 were excluded.

**Results:**

The study population comprised 5.3 million individuals. A total of 54.3% (n=2.9 million) were women, and the mean age was 71.3 (SD 8.16) years. Receiving more than 2 of the 5 recommended screenings and vaccinations examined, compared with receiving none, was associated with a strong reduction in the odds of first-time LTC need (odds ratio [OR] 0.62, 95% CI 0.60‐0.64; *P*<.001). Odds of first-time LTC need were also significantly lower with high numbers of specialist groups and low numbers of specialist days (more than 7 specialist groups consulted and fewer than 48 billing days) compared with no specialist utilization (any specialist utilization: OR 0.82, 95% CI 0.79‐0.85; *P*<.001; more than 7 instead of fewer than 5 specialist groups: OR 0.94, 95% CI 0.92‐0.96; *P*<.001). Likewise, a physiotherapy prescription in between 5 and 10 quarters of the 5-year period instead of none was related to lower odds of first-time LTC need (OR 0.81, 95% CI 0.79‐0.83; *P*<.001). Generalist care, hospitalizations, polypharmacy, and potentially inadequate medication were concomitant with first-time LTC need. Among the disease management programs and medical aids examined, there were both positive and negative relationships with first-time LTC need.

**Conclusions:**

Utilization of recommended screenings, vaccinations, specialist physician care, and physiotherapy is substantially and significantly associated with the nonoccurrence of first-time LTC need in the older German population. Our study provides a foundation for future research on orienting health care toward preventing functional decline.

## Introduction

The growing number of people in need of long-term care (LTC) in aging societies is accompanied by significant challenges for health care systems worldwide. As populations age, the prevalence of chronic diseases, functional impairments, and frailty increases, leading to a steadily rising demand for continuous support and health care services [1]. In Germany, the number of people in need of LTC doubled between 2015 and 2023 to reach 5.7 million. The 2.2% quarterly increase during this period was significantly higher than that projected based on demographic trends [2]. For the purposes of this study, the need for LTC is defined as a condition in which an individual requires significant assistance with activities of daily living and, as a result, successfully applies for benefits under Germany’s statutory LTC insurance scheme [3].

The increasing number of people in need of LTC can be partly attributed to catch-up effects in LTC assessments following the COVID-19 pandemic. Additionally, the so-called “sandwich effect” is under discussion: a simultaneous rise in care-dependent baby boomers and their very old parents. This phenomenon is driven by higher life expectancy and improved survival of individuals with severe illnesses or injuries who require LTC services [4]. This trend is observed not only in Germany but also across industrialized countries worldwide, especially those where population aging is already well advanced, as a recent UN World Social Report states [5]. Although not all older individuals require formal care or support, the overall societal demand for LTC services is rising significantly. In Germany, it is projected that the number of people in need of LTC will rise from 6.0% in 2021 to 9.1% in 2055 [6,7].

Given this trend, the promotion of health and prevention in older age is becoming increasingly important. The goal is to maintain functional health—and thus autonomy and quality of life—for as long as possible, ultimately preventing or delaying the onset of LTC need. Prevention in this context encompasses a wide range of strategies, including health promotion, early detection, and rehabilitative measures. Existing research has largely focused on behavioral preventive approaches aimed at improving physical or cognitive performance among older populations [8]. In contrast, there has been relatively little investigation into structural or health care–related determinants of LTC need [9].

To our knowledge, the role of health care utilization in the years leading up to the onset of LTC need remains an unexplored area, apart from 2 observational studies from Japan, which reported that hyperpolypharmacy, defined as 10 or more medications per month, and prescription of drugs with sedative or anticholinergic properties were associated with an elevated risk of first-time LTC need [10,11]. An ecological Japanese study found that decentralized local prevention services, such as functional training, health education, and support for social activities, significantly reduced the population risk of moderate functional dependency [12]. These findings are specific to the Japanese context and either focus solely on medication or use data at the regional level. The lack of German studies analyzing the association of intersectoral health care utilization with LTC need warrants further individual-level investigation.

This study addresses this research gap by aiming to investigate the associations between the utilization of health care services and first-time LTC need. We analyzed this relationship in individuals aged ≥60 years using linked German statutory health insurance (SHI) and statutory LTC insurance claims data.

## Methods

### Data and Study Design

This retrospective cohort study used anonymized claims data of individuals insured with Germany’s largest SHI and statutory LTC insurance fund, AOK, from 2016 to 2021. In 2024, AOK provided health insurance to one-third of the German population, and 42% of all those in need of LTC were insured with AOK [13]. To qualify as in need of LTC, individuals first must formally apply for LTC benefits. The subsequent LTC need assessment considers both mental and physical limitations to independent living. If 1 of 5 care levels is awarded after the assessment, the person becomes eligible for LTC benefits [3].

During the 5-year exposure period from January 1, 2016, to December 31, 2020, individual health care utilization in terms of outpatient and inpatient physician care, pharmaceutical care, physiotherapy, and medical aids was assessed. Subsequently, individuals were followed for one quarter, from January 1, 2021, to March 31, 2021. In this follow-up period, we determined whether a first-time LTC need was documented. First-time LTC need was dated to the retroactive commencement of services, which often aligns with the application date. To be considered for the study population, individuals had to be aged at least 60 years at the start of the follow-up period. Any individuals with conflicting information regarding date of birth, sex, date of first-time LTC need, or date of death were identified, or whose zip code could not be mapped to regional variables were excluded. LTC need or death before the follow-up period also led to exclusion. We further required continuous insurance coverage and residence in Germany throughout the entire observation period. All individuals meeting the inclusion criteria were included in the study.

### Exposure Variables

#### Physician Care

Variables relating to physician care were defined in terms of care provided by general practitioners (GPs), specialists, inpatient care, screenings and vaccinations, and disease management programs (DMPs). The extent of care provided by GPs was quantified based on the number of days on which a service provided by a GP was documented during the exposure period. Similarly, the extent of specialist care was defined by the number of days with specialist services documented. Additionally, the number of different specialist groups consulted was included as a measure of the broadness of specialist care. Except for urologists, gynecologists, and medical services related to laboratory medicine, all specialist groups were considered for both specialist care variables. Urologists and gynecologists were excluded because, as a relevant part of their services only applies to either sex and the importance of the 2 specialties with respect to LTC need prevention could differ, including them could introduce bias. To quantify the utilization of inpatient care, we determined the frequency of hospitalizations.

The screenings and vaccinations considered were cancer screenings (colon cancer screening through colonoscopy or stool tests, skin cancer screening, and prostate cancer or cervical cancer screening), a general health check-up, and the influenza vaccination. The extent of their utilization was operationalized as the number of screenings and vaccinations that were utilized according to the SHI reimbursement interval (Multimedia Appendix 1). Breast cancer screening was not considered because, until 2024, it was only reimbursed for women aged <70 years. Neither was the pneumococcal vaccination considered, as a single vaccination is recommended for the population aged ≥60 years. If a person had already been vaccinated against pneumococcal diseases before the exposure period, we would have been unable to correctly identify the vaccination.

Regarding structured disease management, we determined whether individuals participated in the established German asthma, chronic obstructive pulmonary disease (COPD), congenital heart disease, and type I or type II diabetes DMPs at any time during the exposure period. German DMPs address all patients with the respective disease rather than focusing on high-risk individuals [14] and promote evidence-based therapy and clinical pathways [15].

#### Pharmaceutical Care

In terms of drug treatment, both polypharmacy and prescription of potentially inappropriate medications (PIMs) that can cause adverse drug events in the older population were included. Polypharmacy was defined as the prescription of 9 or more different drug substances within the same quarter [16]. The definition of PIM was based on whether at least one of the substances from a list of potentially inadequate substances specific to the German context, including analgesics, anti-inflammatory drugs, antiarrhythmic drugs, antibiotics, anticholinergic drugs, antidepressants, antihypertensive agents, neuroleptic drugs, and sedatives, was prescribed [17]. To assess the extent of polypharmacy and PIM, the number of quarters during the exposure period in which an individual’s medication met the defined criteria was computed.

#### Physiotherapy

To assess how often physiotherapy was utilized, the number of quarters with a physiotherapy prescription during the exposure period was counted.

#### Medical Aids

All medical aids that can be reimbursed by SHI in Germany are listed in the catalog of medical aids by the National Association of Statutory Health Insurance Funds [18]. This catalog includes 38 medical aid groups, such as insoles, walking aids, or hearing aids, that were summarized into 6 categories: disease-specific aids, aids supporting self-dependence, orthopedic aids, walking aids, hearing aids, and wheelchairs, including mobility scooters. For each category, we determined whether at least one medical aid from that category was prescribed. The assignment of medical aid groups to the 6 categories is provided in Multimedia Appendix 2.

### Covariates

Comorbidities were included based on the conditions contained in the Elixhauser comorbidity index [19], typical geriatric conditions as defined by Lübke and Meinck [20], and additional diagnosis-based definitions of arthrosis, asthma, chronic obstructive pulmonary disease, and congenital heart disease developed by the AOK Research Institute [21]. The presence of these conditions was determined based on the year before the exposure period. In this way, we ensured that diagnoses were not affected by utilization behavior during the exposure period. Both inpatient and outpatient diagnoses were considered. Outpatient diagnoses were validated by requiring them to be documented as assured (“gesichert” according to the *International Statistical Classification of Diseases and Related Health Problems, 10th revision, German Modification*) in at least two quarters. Multimedia Appendix 3 includes a complete list of the conditions included.

As sociodemographic variables, age, sex, and regional variables were included. We determined the county settlement structure type with the 4 possible outcomes: metropolitan, urban, rural with agglomeration tendency, and rural as defined by the German Federal Institute for Research on Building, Urban Affairs and Spatial Development [22]. The German Index of Socioeconomic Deprivation developed at the Robert Koch Institute measures the level of socioeconomic deprivation based on administrative data on education, employment, and income [23]. It was included at zip code level and ranges from 0 (lowest deprivation) to 1 (highest deprivation).

### Statistical Analysis

Logistic regression was used to assess the association between the variables of interest and first-time LTC need. The outcome data reflect the date when LTC insurance benefits were first claimed, rather than the point of time when a person would have been first eligible, but may not yet have applied for LTC need. Therefore, and given the 3-month follow-up, logistic regression analysis was chosen over using time-to-event methods. First, a crude single exposure model was estimated by univariate regression of first-time LTC need separately on each of the variables of interest. We then controlled for the named comorbidities and sociodemographic variables in an adjusted single exposure model. Finally, all variables of interest were integrated into a single adjusted multiple exposure model, in which covariates were also controlled for.

Owing to the considerable number of variables of interest, whose association with LTC need was tested for significance, we used *P*-values adjusted for multiple testing using a max-*t* test. This test controls the probability of falsely rejecting at least one of the hypotheses in question [24]. All metric variables of interest were categorized into a null category indicating no utilization of the respective service, and 3 categories of low, medium, and high utilization by classification according to the Fisher-Jenks algorithm [25]. Rather than assuming a linear relationship, this flexible approach enabled picking up possible nonlinear and nonmonotonic associations. To be able to estimate the association of the number of specialist billing days and specialist groups simultaneously despite their linear dependence if categorized, we introduced a binary variable displaying whether specialist care was utilized at all and combined the null category and the low utilization category of each of the billing days and specialist groups variable into a single reference category. Data preparation and statistical analysis were conducted using Oracle 19c Enterprise Edition (Oracle Corporation) and R 4.4.2 (R Foundation for Statistical Computing). Statistical significance was evaluated at a 2-sided significance level α of .05.

### Sensitivity Analysis

To assess whether results were driven by effects specific to the observation period and the short duration of follow-up, we first defined 3 alternative observation periods that started on April 1, 2016, July 1, 2016, and October 1, 2016, each also consisting of a 5-year exposure period and one-quarter follow-up period, and second, in another alternative specification, we extended the follow-up period to 1 year. Third, to address the potential for reverse causality, we excluded the last year from the exposure period. For this analysis, we rescaled the utilization category thresholds of each of the exposure variables to reflect the shorter exposure period (Multimedia Appendix 4).

### Ethical Considerations

All analyses described were conducted with the approval of the responsible ethics committee of Charité–Universitätsmedizin Berlin (EA4/147/23), in accordance with national law and the current version of the Declaration of Helsinki. We report our findings in accordance with the STROBE (Strengthening of the Reporting of Observational Studies in Epidemiology) guidelines.

## Results

### Description of the Study Population

**Figure 1.**
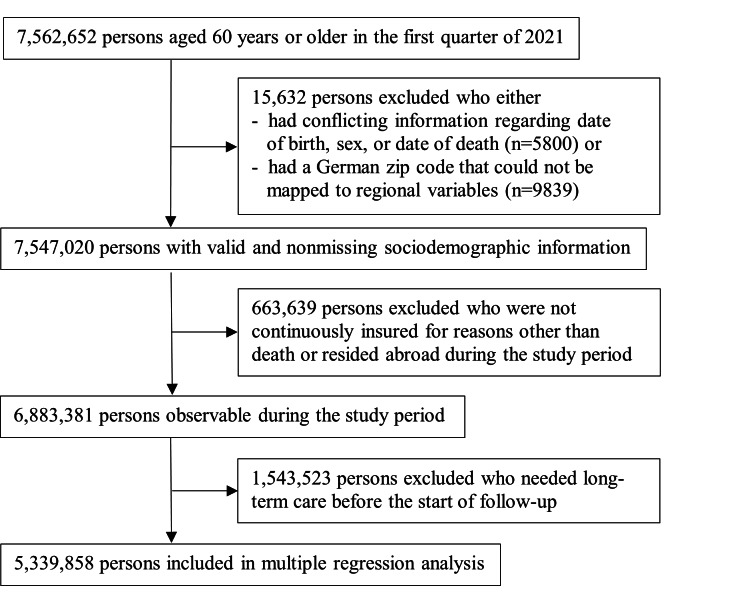
Flowchart of study population selection.

Figure 1 shows the flowchart of study population selection. At the start of the follow-up, 7,562,652 persons aged ≥60 years were insured with AOK. After application of the selection criteria, the final study population comprised 5,339,858 insured persons. Descriptive statistics for the study population are presented in Table 1. The average age was 71.3 (SD 8.16) years, and 54.3% (n=2,899,228) were women. There were no missing values. During the one-quarter follow-up, 91,160 (1.7%) individuals became in need of LTC. See Multimedia Appendices 3-7 for flowcharts and full descriptive statistics, including the sensitivity analysis study populations.

**Table 1.** Descriptive statistics for the study population (n=5,339,858).

Characteristics	Value
Age (y), mean (SD)	71.3 (8.2)
Sex, female, n (%)	2,899,228 (54.3)
German Index of Social Deprivation, mean (SD)	0.5 (0.2)
County settlement structure type, n (%)	
Metropolitan	1,136,388 (21.3)
Urban	1,976,750 (37.0)
Rural with agglomeration tendency	1,186,650 (22.2)
Rural	1,040,070 (19.4)
Utilization of general practitioner (number of days), n (%)	
None (0)	118,747 (2.2)
Low (1-47)	2,568,788 (48.1)
Medium (48-98)	2,159,401 (40.4)
High (>98)	492,922 (9.2)
Utilization of specialist, n (%)	5,053,869 (94.6)
Utilization of specialist (number of groups), n (%)	
Low (0‐4)	2,415,753 (45.2)
Medium (5-7)	1,846,289 (34.6)
High (>7)	1,077,816 (20.2)
Utilization of specialist (number of days), n (%)	
Low (0‐47)	4,259,918 (79.8)
Medium (48-396)	1,075,012 (20.1)
High (>396)	4928 (0.1)
Hospitalizations, n (%)	
None (0)	2,594,401 (48.6)
Low (1-2)	1,889,121 (35.4)
Medium (3-6)	734,973 (13.8)
High (>6)	121,363 (2.3)
Screenings and vaccinations (number of services), n (%)	
None (0)	852,863 (16.0)
Low (1)	1,704,527 (31.9)
Medium (2)	1,513,223 (28.3)
High (>2)	1,269,245 (23.8)
DMP^a^ congenital heart disease enrollment, n (%)	531,093 (9.9)
DMP chronic obstructive pulmonary disease enrollment, n (%)	218,486 (4.1)
DMP asthma enrollment, n (%)	146,291 (2.7)
DMP diabetes enrollment, n (%)	1,113,656 (20.9)
Polypharmacy (number of quarters), n (%)	
None (0)	4,425,424 (82.9)
Low (1-4)	617,878 (11.6)
Medium (5-11)	193,477 (3.6)
High (>11)	103,079 (1.9)
Prescription of potentially inadequate medications (number of quarters), n (%)	
None (0)	3,669,247 (68.7)
Low (1-4)	1,136,815 (21.3)
Medium (5-12)	282,097 (5.3)
High (>12)	251,699 (4.7)
Physiotherapy (number of quarters), n (%)	
None (0)	2,559,641 (47.9)
Low (1-4)	2,005,639 (37.6)
Medium (5-10)	578,229 (10.8)
High (>10)	196,349 (3.7)
Orthopedic aids prescription, n (%)	2,259,257 (42.3)
Hearing aids prescription, n (%)	567,833 (10.6)
Walking aids prescription, n (%)	593,506 (11.1)
Wheelchairs including mobility scooters prescription, n (%)	57,821 (1.1)
Aids supporting self-dependence prescription, n (%)	853,723 (16.0)
Disease-specific aids prescription, n (%)	1,622,125 (30.4)

a Disease management program.

### Regression Analysis

The odds ratios (ORs) resulting from estimating association of health care utilization with first-time need for LTC are presented in Table 2. The results reported in the following sections refer to the adjusted multiple exposure model.

**Table 2.** Results of logistic regression of first-time long-term care need on health care utilization variables.

Variables	Crude single exposure model		Adjusted single exposure model^a^		Adjusted multiple exposure model^a^	
	Odds ratio (95% CI)	*P* value	Odds ratio (95% CI)	*P* value	Odds ratio (95% CI)	*P* value
Utilization of general practitioner (number of billing days)						
None (0)	Reference	—^b^	Reference	—	Reference	—
Low (1-47)	1.28 (1.20‐1.37)	<.001	1.26 (1.18‐1.34)	<.001	1.38 (1.29‐1.48)	<.001
Medium (48-98)	2.65 (2.49‐2.83)	<.001	1.65 (1.55‐1.76)	<.001	1.59 (1.48‐1.71)	<.001
High (>98)	4.35 (4.08‐4.65)	<.001	2.16 (2.02‐2.31)	<.001	1.65 (1.53‐1.77)	<.001
Utilization of specialist						
None (0 groups and billing days)	Reference	—	Reference	—	Reference	—
Positive number of groups (>0) and billing days (>0)	1.12 (1.08‐1.16)	<.001	0.95 (0.92‐0.98)	<.001	0.82 (0.79‐0.85)	<.001
Utilization of specialist (number of specialist groups)						
Low (0‐4)	Reference	—	Reference	—	Reference	—
Medium (5-7)	1.03 (1.02‐1.05)	<.001	1.03 (1.02‐1.05)	<.001	0.95 (0.93‐0.96)	<.001
High (>7)	1.02 (1.00‐1.04)	.07	1.12 (1.09‐1.14)	<.001	0.94 (0.92‐0.96)	<.001
Utilization of specialist (number of billing days)						
Low (0‐47)	Reference	—	Reference	—	Reference	—
Medium (48-396)	1.52 (1.50‐1.55)	<.001	1.20 (1.18‐1.22)	<.001	1.06 (1.04‐1.08)	<.001
High (>396)	3.37 (2.96‐3.82)	<.001	3.09 (2.70‐3.53)	<.001	1.70 (1.48‐1.95)	<.001
Hospitalizations						
None (0)	Reference	—	Reference	—	Reference	—
Low (1-2)	1.96 (1.93‐1.99)	<.001	1.62 (1.59‐1.65)	<.001	1.46 (1.44‐1.49)	<.001
Medium (3-6)	3.47 (3.41‐3.53)	<.001	2.55 (2.50‐2.60)	<.001	2.00 (1.96‐2.05)	<.001
High (>6)	5.98 (5.82‐6.15)	<.001	4.53 (4.40‐4.67)	<.001	3.07 (2.97‐3.17)	<.001
Screenings and vaccinations (number of services)						
None (0)	Reference	—	Reference	—	Reference	—
Low (1)	1.11 (1.09‐1.13)	<.001	0.94 (0.93‐0.96)	<.001	0.90 (0.88‐0.92)	<.001
Medium (2)	1.08 (1.06‐1.10)	<.001	0.84 (0.82‐0.86)	<.001	0.77 (0.76‐0.79)	<.001
High (>2)	0.91 (0.89‐0.93)	<.001	0.68 (0.66‐0.69)	<.001	0.62 (0.60‐0.64)	<.001
DMP^c^ congenital heart disease enrollment	1.74 (1.70‐1.77)	<.001	0.98 (0.95‐1.00)	.09	0.93 (0.91‐0.96)	<.001
DMP chronic obstructive pulmonary disease enrollment	1.66 (1.62‐1.70)	<.001	1.24 (1.19‐1.28)	<.001	1.19 (1.14‐1.23)	<.001
DMP asthma enrollment	1.05 (1.01‐1.09)	.03	0.96 (0.91‐1.01)	.11	0.98 (0.93‐1.03)	>.99
DMP diabetes enrollment	1.68 (1.65‐1.70)	<.001	1.06 (1.03‐1.09)	<.001	0.99 (0.96‐1.02)	>.99
Polypharmacy (number of quarters)						
None (0)	Reference	—	Reference	—	Reference	—
Low (1-4)	2.58 (2.54‐2.62)	<.001	1.88 (1.85‐1.91)	<.001	1.38 (1.35‐1.40)	<.001
Medium (5-11)	3.37 (3.29‐3.45)	<.001	2.30 (2.24‐2.36)	<.001	1.49 (1.45‐1.53)	<.001
High (>11)	4.09 (3.97‐4.21)	<.001	2.84 (2.75‐2.94)	<.001	1.68 (1.62‐1.74)	<.001
Prescription of potentially inadequate medications (number of quarters)						
None (0)	Reference	—	Reference	—	Reference	—
Low (1-4)	1.38 (1.36‐1.40)	<.001	1.27 (1.25‐1.29)	<.001	1.08 (1.07‐1.10)	<.001
Medium (5-12)	1.86 (1.81‐1.91)	<.001	1.44 (1.40‐1.47)	<.001	1.11 (1.08‐1.14)	<.001
High (>12)	2.18 (2.13‐2.24)	<.001	1.42 (1.39‐1.46)	<.001	1.14 (1.11‐1.17)	<.001
Physiotherapy (number of quarters)						
None (0)	Reference	—	Reference	—	Reference	—
Low (1-4)	1.00 (0.99‐1.01)	>.99	1.01 (1.00‐1.03)	0.11	0.85 (0.84‐0.86)	<.001
Medium (5-10)	1.16 (1.14‐1.19)	<.001	1.13 (1.10‐1.15)	<.001	0.81 (0.79‐0.83)	<.001
High (>10)	1.61 (1.57‐1.66)	<.001	1.42 (1.37‐1.46)	<.001	0.98 (0.95‐1.01)	>.99
Orthopedic aids prescription	1.09 (1.07‐1.10)	<.001	1.11 (1.09‐1.12)	<.001	0.94 (0.93‐0.96)	<.001
Hearing aids prescription	2.01 (1.97‐2.04)	<.001	1.01 (0.99‐1.02)	0.44	0.98 (0.96‐0.99)	0.22
Walking aids prescription	3.66 (3.61‐3.71)	<.001	2.13 (2.09‐2.16)	<.001	1.62 (1.59‐1.64)	<.001
Wheelchairs including mobility scooters prescription	3.84 (3.71‐3.98)	<.001	2.38 (2.30‐2.47)	<.001	1.43 (1.38‐1.49)	<.001
Aids supporting self-dependence prescription	2.84 (2.80‐2.88)	<.001	1.80 (1.77‐1.83)	<.001	1.31 (1.29‐1.33)	<.001
Disease-specific aids prescription	1.67 (1.65‐1.70)	<.001	1.43 (1.41‐1.45)	<.001	1.14 (1.12‐1.16)	<.001

a Adjusted for comorbidities, age, sex, county settlement structure type, and German Index of Social Deprivation.

b Not applicable.

c DMP: disease management program.

#### Physician Care

Regarding outpatient physician care, a low number of GP visits was related to a significant increase in the odds of first-time LTC need (OR 1.38, 95% CI 1.29‐1.48; *P*<.001) relative to none. Odds for first-time LTC need rose further in medium (OR 1.59, 95% CI 1.48‐1.71; *P*<.001) and high levels of utilization (OR 1.65, 95% CI 1.53‐1.77; *P*<.001). With a low number of specialist groups and billing days, utilization of specialist care entailed a reduction of odds versus no utilization (OR 0.82, 95% CI 0.79‐0.85; *P*<.001). Compared to this low utilization level, odds reduced further in medium (OR 0.95, 95% CI 0.93‐0.96; *P*<.001) and high (OR 0.94, 95% CI 0.92‐0.96; *P*<.001) numbers of specialist groups but increased in medium (OR 1.06, 95% CI 1.04‐1.08; *P*<.001) and high (OR 1.70, 95% CI 1.48‐1.95; *P*<.001) numbers of specialist days. Hospitalizations were strongly and significantly associated with a higher risk of first-time LTC need. More than 6 hospitalizations within the 5-year exposure period were related to a more than 3-fold increase in the odds of first-time LTC need (OR 3.07, 95% CI 2.97‐3.17; *P*<.001) relative to none.

A high utilization of screenings and vaccinations was related to a reduction in the odds of first-time LTC need (OR 0.62, 95% CI 0.60‐0.64; *P*<.001). Although less strong in magnitude, low (OR 0.90, 95% CI 0.88‐0.92; *P*<.001) and medium (OR 0.62, 95% CI 0.60‐0.64; *P*<.001) utilization of screenings and vaccinations were also significantly negatively associated with first-time LTC need. Of the 4 DMPs, only the congenital heart disease DMP exhibited a significant protective relationship with first-time LTC need (OR 0.93, 95% CI 0.91‐0.96; *P*<.001). COPD DMP participation predicted an increased risk of first-time LTC need (OR 1.19, 95% CI 1.14‐1.23; *P*<.001).

#### Pharmaceutical Care

Both prescription of PIM and polypharmacy were accompanied by higher odds of first-time LTC need. The relationship is stronger in magnitude for high levels of polypharmacy (OR 1.68, 95% CI 1.62‐1.74; *P*<.001) than for high levels of PIM (OR 1.14, 95% CI 1.11‐1.17; *P*<.001).

#### Physiotherapy

Relative to nonuse, a low (OR 0.85, 95% CI 0.84‐0.86; *P*<.001) or medium (OR 0.81, 95% CI 0.79‐0.83; *P*<.001) utilization of physiotherapy correlated negatively with first-time LTC need. With a high utilization level, the association did not remain statistically significant (*P*>.99).

#### Medical Aids

Orthopedic aids had a significant negative association with first-time LTC need (OR 0.94, 95% CI 0.93‐0.96; *P*<.001). Walking aids (OR 1.62, 95% CI 1.59‐1.64; *P*<.001), wheelchairs including mobility scooters (OR 1.43, 95% CI 1.38‐1.49; *P*<.001), aids supporting self-dependence (OR 1.31, 95% CI 1.29‐1.33; *P*<.001), and disease-specific aids (OR 1.14, 95% CI 1.12‐1.16; *P*<.001) were related to an increased risk of first-time LTC need.

### Sensitivity Analysis

As shown in Multimedia Appendix 8, the 3 alternative observation periods yielded similar results compared to the main analysis. Multimedia Appendix 9 presents the results obtained when extending the follow-up period to 1 year. While most associations exhibited no notable difference to the main analysis, the relationship between LTC need and the number of hospitalizations became weaker, and the negative associations of the DMPs for asthma (OR 0.95, 95% CI 0.92‐0.98; *P*=.008) and diabetes (OR 0.95, 95% CI 0.93‐0.96; *P*<.001) with LTC need became significant. Omitting the final year of the exposure period, as displayed in Multimedia Appendix 10, produced analogous estimates but for the number of hospitalizations. As when extending the follow-up duration to 1 year, hospitalizations displayed lower estimates for all utilization categories. Relative to the main analysis, the OR of more than 6 hospitalizations (OR 1.80, 95% CI 1.74‐1.86; *P*<.001) was almost halved.

## Discussion

### Principal Findings

We have shown that a wide range of measures of health care utilization display significant and strong associations with the risk of first-time LTC need. To the best of our knowledge, our study is the first to analyze the interplay of the frequency of physician visits, DMP enrollment, physiotherapy utilization, prescription of medical aids, screenings and vaccinations, and inadequate medication supply with first-time LTC need simultaneously. In the population aged ≥60 years, utilization of specialist care, physiotherapy, and screenings and vaccinations display the strongest relationship with nonoccurrence of first-time LTC need. At the same time, frequent utilization of GP care, hospitalizations, polypharmacy, and prescription of walking aids showed a significant association with first-time LTC need. As this study relied on a 5-year exposure period to evaluate health care utilization, the relationship between health care use in a 5-year period and LTC need can only be assessed in and does only apply to individuals who neither need LTC nor die within 5 years. This approach provides a longer-term perspective on the health care trajectory of a person, which may be more meaningful for prevention than a single event.

### Comparison With Prior Work

Apart from the 2 studies, which corroborated that inadequate medication supply is related to an increase in future LTC risk [10,11], further evidence on the relationship of health care utilization with first-time LTC need is missing. LTC need being a sociolegal concept in Germany that differs substantially from that in other countries [3,26], prior research has focused on related outcomes, such as frailty, functional measures, morbidity, or mortality [8,27,28]. Although these outcomes must be distinguished both from each other and first-time LTC need, they are interrelated [29-32] and evidence on how they correlate with health care utilization can promote our understanding of the link between health care utilization and first-time LTC need.

#### Frequency of Physician Contact

We evaluated 3 variables that are closely related to the frequency of physician contact: GP billing days, specialist physician billing days, and hospital admissions. Compared to no contact, both GP billing days and hospital admissions were related to an increased risk of first-time LTC need. While utilization of specialist care with a low number of billing days and specialist groups showed a preventive association, all 3 variables have in common that compared to a low level of utilization, a higher level of physician contact was related to an increase in the risk of first-time LTC need. On the one hand, frequent physician contact may entail constant assessment of health risks and subsequent counseling and thereby exerting a preventive effect on LTC need risk [33,34]. On the other hand, there is a wealth of literature on how physical frailty is associated with a higher utilization of health care in terms of outpatient care, inpatient care, home care, and nursing home admission [35-40]. Moreover, individuals with an adverse status in terms of living alone, social relations, and social support, referred to as socially frail, are reported to have a lower number of physician visits and lower likelihood of hospitalization, hinting at lower ability to access health care services including LTC [41]. Despite our efforts to control for comorbidities, both physical and social frailty likely act as confounders, correlating both with first-time LTC need risk and health care utilization.

In sensitivity analyses that excluded the last year from the exposure period or extended follow-up to 1 year, we also observed a markedly weaker association between hospitalizations and LTC need. This is consistent with the fact that hospitalizations frequently occur shortly before first-time LTC need [42]. If there is a time lag between the end of the exposure period and the date of first-time LTC need, hospitalizations shortly before first-time LTC need will not be reflected in the exposure variable. This underscores how the strong relationship between hospitalizations and LTC need in the main analysis reflects morbidity levels. In summary, while the frequency of physician contact is a valuable identifier of persons at risk of first-time LTC need, it cannot be assumed to be detrimental to independent living based on our analysis.

#### Condition-Specific Health Care Utilization

Apart from the frequency of physician contact, we also evaluated how extensive specialist outpatient care utilization was by including the number of specialist groups consulted. At a constant number of specialist billing days, an increase in specialist groups consulted was connected to a decreased risk of first-time LTC need. Similarly, another claims data–based study from Germany reported that, adjusted for GP care utilization, LTC beneficiaries are less likely to visit physicians from different specialist groups than older people without LTC need [43]. We cannot rule out that part of the promising association we estimated pertains to barriers to accessibility of specialist care. These might be more pronounced for individuals at higher risk of first-time LTC need. Still, hypothesizing that even with few patient-physician contacts, each specialist group can give specific preventive recommendations and guidance, it is plausible that a wider variety of specialist groups consulted, holding the number of billing days fixed, exerts a preventive effect. On the other hand, having visited a given specialist more frequently may reflect higher morbidity levels more so than an improved capacity to provide preventive guidance.

Of the 4 DMPs evaluated, we found a preventive relationship consistent across main and sensitivity analyses regarding only the congenital heart disease DMP, while the COPD DMP even showed a detrimental association. As we can only account for disease presence and not severity, selection effects might bias our estimates if DMP participants differ from nonparticipants in this regard. DMPs have not yet been evaluated in terms of their association with first-time LTC need, nor have there been DMP evaluations using randomized control trials in Germany [44].

While we found physiotherapy utilization to be predictive of nonoccurrence of first-time LTC need, when receiving prescriptions in up to 10 quarters within 5 years, we saw no such association for higher levels of utilization. There is prior evidence that physiotherapy can support the use of medical aids [45], prevent institutionalization after hospitalization [46], and, in the case of a Spanish study, reduce mortality through improvement in quality of life, alleviation of musculoskeletal disorders, a referral function in the health care system, and fall prevention [47]. While there is currently no evidence on the relationship between physiotherapy utilization and first-time LTC need, 2 articles report that frail persons receive physiotherapy more often than nonfrail ones [36,38].

Furthermore, our medical aids analysis showed that only orthopedic devices had a negative relationship with first-time LTC need. All other categories except for hearing aids had a positive association that was most pronounced in walking aids. A meta-analysis suggested that insoles, which make up for the largest share of prescriptions within the orthopedic devices group, were beneficial for balance improvement and fall prevention [48], supporting our finding of a preventive potential. Although walking aids are intended to prevent falls, walking aid users face a high risk of falls and injuries that might be reduced by improvements in devices and better instructions on device usage [45,49]. Likely, both difficulties in accounting for confounders such as gait quality and potential for improvement in walking aid use contribute to the strong positive association with first-time LTC need we observe.

#### Screenings and Vaccinations

The cancer screenings, general health check, and influenza vaccination summarized in a single variable displayed a strong relationship with nonoccurrence of first-time LTC need. There is tentative evidence supporting the health benefits of these services in the population aged ≥60 years, yet the association we find remains high against this backdrop.

Despite the potential of cancer screening to reduce all-cause mortality by 1% to 3% in Germany [50], screening cessation may be recommended for prostate cancer, cervical cancer, and colorectal cancer. With increasing age and decreasing life expectancy, potential harms in screening may outweigh potential benefits, necessitating an individualized screening decision [51-53]. On the one hand, this calls into question whether cancer screenings can have a substantial preventive effect in terms of LTC need. On the other hand, it raises the question of whether part of the observed effect reflects screening cessation recommendations being put into practice. General health checks and health risk assessments have been shown to increase uptake of other preventive services, but an effect on mortality cannot be substantiated despite positive impact on chronic diseases treatment and patient-reported outcomes [34,54-56]. The influenza vaccination provides the most concrete reference for a potential delaying effect on first-time LTC need. Not only is it crucial to influenza prevention, especially so in older adults [57], but it is also related to a decreased risk of dementia [58,59], the third most-named diagnosis for first-time LTC need in Germany in 2021 [60].

Other than effects of screenings and vaccinations themselves, preventive health behavior could contribute to the strength of the observed association. It has been shown that the uptake of preventive services is higher in individuals with health-promoting behavior [61,62]. As we have no information on crucial behavioral aspects such as physical activity, nutrition, and alcohol and tobacco use, they likely bias the observed relationship toward prevention. Bearing that in mind, our study provides first hints for a possible delaying effect of screenings and vaccinations on first-time LTC need that further studies should investigate in more detail on a broader base of data.

#### Inadequate Medication Supply

Our finding that inadequate medication supply is positively related to first-time LTC need and polypharmacy, more so than PIM, is in line with prior cross-sectional [63] and longitudinal studies [10,11] from Japan. Kuroda et al [10] evaluated polypharmacy and PIM using drug prescriptions from a 2-year exposure period and reported associations of similar magnitude. Inadequate medication supply has been shown to be associated with falls, dementia, functional and cognitive decline, and sarcopenia [28,64,65], all of which are plausible causes of first-time LTC need. This suggests that correction of inadequate medication supply can be a leverage point to LTC need prevention.

### Strengths and Limitations

This retrospective cohort study adheres to certain conditions necessary to justify causal statements about the observed associations. Outcome was evaluated after exposure, and thus, reverse causality through effects of utilization of LTC insurance services can be ruled out. Still, utilization might be driven by morbidity trends on a trajectory approaching first-time LTC need [42]. Excluding the final year of the exposure period from the analysis showed that only estimates related to hospitalization are affected strongly by such morbidity trends. The robustness of the remaining estimates indicates that reverse causality through short-term morbidity trends is no major concern. The large nationwide sample enabled us to assess utilization of multiple health care services simultaneously despite their interrelation. In this way, we were able to measure the relationship of each utilization variable with first-time LTC need given the utilization pattern in terms of the other variables.

Nonetheless, this study faces some substantial limitations that confine the results to the domain of correlation. It is the nature of claims data that they lack information on disease severity, health-promoting behavior, social networks, and individual-level socioeconomic variables. These parameters need to be integrated to enable attainment of unbiased estimates, given that they are related to physical and social frailty, as well as the accessibility of health care [66,67]. Furthermore, despite the large sample size, results attained based on AOK-insured individuals cannot be generalized to the German population, as they tend to have lower socioeconomic status and higher comorbidity burden than people insured privately or with other SHI funds [68,69]. On top of that, our observation period coincides with the COVID-19 pandemic, whose effect on health care utilization limits the external temporal validity of our study.

Moreover, a range of health care services that may contribute to LTC need prevention, such as breast cancer screening, geriatric assessment, and rehabilitation, were not evaluated as part of this study. This study was designed to estimate associations of health care utilization with first-time LTC need without placing further restrictions on the study population than the criterion of being aged ≥60 years. Therefore, services such as breast cancer screening and geriatric assessment, access to which is specific to sex and age, could not be included. In Germany, rehabilitation services are covered by the statutory pension insurance scheme before retirement. Utilization of rehabilitation services can thus not be assessed on SHI claims data alone for a population partially still in employment. Furthermore, we primarily aimed to assess the association of health care utilization with first-time LTC need as a proxy for functional health. We must concede that claiming LTC benefits is itself an act of health care utilization that, besides functional health, is determined by access to health care and utilization behavior. As a determinant of health care accessibility and utilization behavior, individual-level socioeconomic variables could be a valuable addition in factoring out its confounding impact.

### Conclusions

In conclusion, we have shown that the utilization of recommended screenings, vaccinations, specialist physician care, and physiotherapy displays substantial and significant associations with nonoccurrence of first-time LTC need in the German population aged ≥60 years. Our study provides an important foundation for future research. It is essential to address the limitations posed by unobserved confounding variables and the nonexperimental study design to generate evidence on how LTC need can be delayed or avoided. Such evidence could provide guidance on orienting health care toward preventing functional decline.

## Supplementary material

10.2196/86572 Multimedia Appendix 1 Definition of the screenings and vaccinations utilization variable.

10.2196/86572 Multimedia Appendix 2 Assignment of medical aid groups to medical aid categories.

10.2196/86572 Multimedia Appendix 3 Descriptive statistics of study population in main analysis and sensitivity analyses 1 to 3.

10.2196/86572 Multimedia Appendix 4 Descriptive statistics for study population in sensitivity analysis 5.

10.2196/86572 Multimedia Appendix 5 Flowchart of study population selection for sensitivity analysis 1.

10.2196/86572 Multimedia Appendix 6 Flowchart of study population selection for sensitivity analysis 2.

10.2196/86572 Multimedia Appendix 7 Flowchart of study population selection for sensitivity analysis 3.

10.2196/86572 Multimedia Appendix 8 Results of logistic regression of first-time long-term care need on health care utilization variables in main analysis and sensitivity analyses 1 to 3.

10.2196/86572 Multimedia Appendix 9 Results of logistic regression of first-time long-term care need on health care utilization variables in main analysis and sensitivity analysis 4.

10.2196/86572 Multimedia Appendix 10 Results of logistic regression of first-time long-term care need on health care utilization variables in main analysis and sensitivity analysis 5.

## Abbreviations

COPD: chronic obstructive pulmonary disease
DMP: disease management program
GP: general practitioner
LTC: long-term care
OR: odds ratio
PIM: potentially inappropriate medications
SHI: statutory health insurance
STROBE: Strengthening of the Reporting of Observational Studies in Epidemiology
