# Supplementary material for: Health Care–Related Determinants of First-Time Long-Term Care Need in Older Adults in Germany: Retrospective Cohort Study Using Claims Data
Source: Interact J Med Res. 2026 Jul 20;15:e86572. doi: 10.2196/86572 (PMC13384046; doi:10.2196/86572)
Supplement: Multimedia Appendix 1 [file ijmr-v15-e86572-s001.docx]

|  | Maximum utilization interval | Threshold to be considered utilized in the five-year exposure observation period | Billing codes according to Einheitlicher Bewertungsmaßstab [1] |
| --- | --- | --- | --- |
| Colon cancer screening |  |  |  |
| Colonoscopy | Ten years apart, if for screening purpose | Once | 01741, 13421 and regional codes |
| Stool test | Every other year, if no colonoscopy is provided | Twice | 01734, 01738, 32040, 32457 and regional codes |
| Skin cancer screening | Every other year | Twice | 01745, 01746 and regional codes |
| Prostate cancer or cervical cancer screening |  |  |  |
| Prostate cancer screening | Yearly | Five times | 01731 and regional codes |
| Cervical cancer screening by Pap test | Yearly | Five times | 01730, 01760, 01761 and regional codes |
| General health check | Every three years | Once | 01732 and regional codes |
| Influenza vaccination | Yearly | Five times | 89111, 89112 and regional codes |

Note: Colon cancer screening was considered utilized if either a one colonscopy was performed or two stool tests were made. Prostate cancer and cervical cancer screening were pooled and were understood as an indicator of whether yearly sex-specific cancer screening was utilized. All services were accessible to everyone within the study population regarding age restrictions on reimbursement by statutory health insurance in Germany [2].

1. Einheitlicher Bewertungsmaßstab. Kassenärztliche Bundesvereinigung. 2025. URL: <https://www.kbv.de/praxis/abrechnung/ebm> [Accessed 2025-09-05]

2. Früherkennungsmonitor 2024: Inanspruchnahme von Krebs-Früherkennungsleistungen der GKV. Dräther H, Zok K, Eymers E, Schillinger G. 2024. URL: <https://www.wido.de/fileadmin/Dateien/Dokumente/Forschung_Projekte/Ambulante_Versorgung/wido_frueherkennungsmonitor_2024_final.pdf> [Accessed 2025-08-11]
