# Supplementary material for: Health Care–Related Determinants of First-Time Long-Term Care Need in Older Adults in Germany: Retrospective Cohort Study Using Claims Data
Source: Interact J Med Res. 2026 Jul 20;15:e86572. doi: 10.2196/86572 (PMC13384046; doi:10.2196/86572)
Supplement: Multimedia Appendix 2 [file ijmr-v15-e86572-s002.docx]

|  | Medical aid group | Medical aid group number |
| --- | --- | --- |
| Disease-specific aids | Suction devices | 01 |
|  | Radiation therapy devices | 06 |
|  | Aids for the blinds | 07 |
|  | electro therapy devices | 09 |
|  | Anti-decubitus aids | 11 |
|  | Tracheostomy and laryngectomy aids | 12 |
|  | Inhalation and respiratory therapy devices | 14 |
|  | Compression therapy devices | 17 |
|  | Positioning aids | 20 |
|  | Diagnostic measurement devices | 21 |
|  | Leg prostheses | 24 |
|  | Stoma care products | 29 |
|  | Glucose management devices | 30 |
|  | Therapeutic exercise devices | 32 |
|  | Wigs | 34 |
|  | Epitheses | 35 |
|  | Ocular prostheses | 36 |
|  | Breast protheses | 37 |
|  | Arm prostheses | 38 |
| Devices for independent living | Dressing aids (e.g., sock pullers) | 02 |
|  | Application aids | 04 |
|  | Bath and shower aids | 04 |
|  | Incontinence aids | 15 |
|  | Communication aids | 16 |
|  | Mobility aids | 22 |
|  | Visual aids | 25 |
|  | Seating aids | 26 |
|  | Speech aids | 27 |
|  | Standing aids | 28 |
|  | Toilet aids | 33 |
| Orthopedic aids | Bandages | 05 |
|  | Insoles | 08 |
|  | Ortheses/Splints | 23 |
|  | Orthopedic shoes | 31 |
| Walking aids | Walking aids | 10 |
| Hearing aids | Hearing aids | 13 |
| Wheelchairs including mobility scooters | Wheelchairs and disability vehicles | 18 |

Note: Medical aid group numbers refer to the enumaration in the catalogue of medical aids by the National Association of Statutory Health Insurance Funds [1]

1. Medical aids list. National Association of Statutory Health Insurance Funds. 2025. URL: <https://hilfsmittel.gkv-spitzenverband.de/home> [Accessed 2025-08-11]
