# Supplementary material for: Health Care–Related Determinants of First-Time Long-Term Care Need in Older Adults in Germany: Retrospective Cohort Study Using Claims Data
Source: Interact J Med Res. 2026 Jul 20;15:e86572. doi: 10.2196/86572 (PMC13384046; doi:10.2196/86572)
Supplement: Multimedia Appendix 3 [file ijmr-v15-e86572-s003.docx]

|  | Main analysis study population (n=5,339,858) | Sensitivity analysis 1 study population (n=5,335,365) | Sensitivity analysis 2 study population (n=5,322,809) | Sensitivity analysis 3 study population (n=5,310,929) |
| --- | --- | --- | --- | --- |
| Age in years, mean (SD) | 71.3 (8.2) | 71.2 (8.1) | 71.1 (8.1) | 71.1 (8.1) |
| Sex, n (%) |  |  |  |  |
| Female | 2,899,228 (54.3) | 2,892,346 (54.2) | 2,881,105 (54.1) | 2,870,930 (54.1) |
| Male | 2,440,630 (45.7) | 2,443,019 (45.8) | 2,441,704 (45.9) | 2,439,999 (45.9) |
| German Index of Social Deprivation, mean (SD) | 0.5 (0.2) | 0.5 (0.2) | 0.5 (0.2) | 0.5 (0.2) |
| County settlement structure type, n (%) |  |  |  |  |
| Metropolitan | 1,136,388 (21.3) | 1,134,466 (21.3) | 1,129,913 (21.2) | 1,125,654 (21.2) |
| Urban | 1,976,750 (37.0) | 1,985,933 (37.2) | 1,980,654 (37.2) | 1,976,851 (37.2) |
| Rural with agglomeration tendency | 1,186,650 (22.2) | 1,133,370 (21.2) | 1,132,124 (21.3) | 1,130,302 (21.3) |
| Rural | 1,040,070 (19.5) | 1,081,596 (20.3) | 1,080,118 (20.3) | 1,078,122 (20.3) |
| Utilization of general practitioner (number of days), n (%) |  |  |  |  |
| None (=0) | 118,747 (2.2) | 118,756 (2.2) | 107,802 (2.0) | 102,387 (1.9) |
| Low (1–47) | 2,568,788 (48.1) | 2,590,880 (48.6) | 2,609,872 (49.0) | 2,619,837 (49.3) |
| Medium (48–98) | 2,159,401 (40.4) | 2,143,885 (40.2) | 2,134,964 (40.1) | 2,127,516 (40.1) |
| High (>98) | 492,922 (9.2) | 481,844 (9.0) | 470,171 (8.8) | 461,189 (8.7) |
| Utilization of specialist, n (%) |  |  |  |  |
| No | 285,989 (5.4) | 289,087 (5.4) | 291,942 (5.5) | 292,180 (5.5) |
| Yes | 5,053,869 (94.6) | 5,046,278 (94.6) | 5,030,867 (94.5) | 5,018,749 (94.5) |
| Utilization of specialist (number of groups), n (%) |  |  |  |  |
| Low (0–4) | 2,415,753 (45.2) | 2,426,517 (45.5) | 2,437,007 (45.8) | 2,435,627 (45.9) |
| Medium (5–7) | 1,846,289 (34.6) | 1,839,563 (34.5) | 1,829,456 (34.4) | 1,823,014 (34.3) |
| High (>7) | 1,077,816 (20.2) | 1,069,285 (20.0) | 1,056,346 (19.8) | 1,052,288 (19.8) |
| Utilization of specialist (number of days), n (%) |  |  |  |  |
| Low (0–47) | 4,259,918 (79.8) | 4,269,340 (80.0) | 4,273,241 (80.3) | 4,270,516 (80.4) |
| Medium (48–396) | 1,075,012 (20.1) | 1,061,207 (19.9) | 1,044,805 (19.6) | 1,035,773 (19.5) |
| High (>396) | 4,928 (0.1) | 4,818 (0.1) | 4,763 (0.1) | 4,640 (0.1) |
| Hospitalizations, n (%) |  |  |  |  |
| None (=0) | 2,594,401 (48.6) | 2,626,378 (49.2) | 2,643,707 (49.7) | 2,649,463 (49.9) |
| Low (1–2) | 1,889,121 (35.4) | 1,879,006 (35.2) | 1,868,594 (35.1) | 1,862,434 (35.1) |
| Medium (3–6) | 734,973 (13.8) | 714,580 (13.4) | 699,580 (13.1) | 690,857 (13.0) |
| High (>6) | 121,363 (2.3) | 115,401 (2.2) | 110,928 (2.1) | 108,175 (2.0) |
| Screenings and vaccinations (number of services), n (%) |  |  |  |  |
| None (=0) | 852,863 (16.0) | 872,862 (16.4) | 883,793 (16.6) | 895,736 (16.9) |
| Low (=1) | 1,704,527 (31.9) | 1,743,480 (32.7) | 1,761,743 (33.1) | 1,785,476 (33.6) |
| Medium (=2) | 1,513,223 (28.3) | 1,529,943 (28.7) | 1,522,991 (28.6) | 1,511,958 (28.5) |
| High (>2) | 1,269,245 (23.8) | 1,189,080 (22.3) | 1,154,282 (21.7) | 1,117,759 (21.0) |
| DMP congenital heart disease enrolment, n (%) |  |  |  |  |
| No | 4,808,765 (90.1) | 4,808,738 (90.1) | 4,803,389 (90.2) | 4,796,895 (90.3) |
| Yes | 531,093 (9.9) | 526,627 (9.9) | 519,420 (9.8) | 514,034 (9.7) |
| DMP chronic obstructive pulmonary disease enrolment, n (%) |  |  |  |  |
| No | 5,121,372 (95.9) | 5,118,541 (95.9) | 5,108,980 (96.0) | 5,099,693 (96.0) |
| Yes | 218,486 (4.1) | 216,824 (4.1) | 213,829 (4.0) | 211,236 (4.0) |
| DMP asthma enrolment, n (%) |  |  |  |  |
| No | 5,193,567 (97.3) | 5,188,001 (97.2) | 5,175,323 (97.2) | 5,163,104 (97.2) |
| Yes | 146,291 (2.7) | 147,364 (2.8) | 147,486 (2.8) | 147,825 (2.8) |
| DMP diabetes enrolment, n (%) |  |  |  |  |
| No | 4,226,202 (79.1) | 4,225,357 (79.2) | 4,221,075 (79.3) | 4,214,870 (79.4) |
| Yes | 1,113,656 (20.9) | 1,110,008 (20.8) | 1,101,734 (20.7) | 1,096,059 (20.6) |
| Polypharmacy (number of quarters), n (%) |  |  |  |  |
| None (=0) | 4,425,424 (82.9) | 4,434,838 (83.1) | 4,435,999 (83.3) | 4,430,458 (83.4) |
| Low (1–4) | 617,878 (11.6) | 609,072 (11.4) | 599,889 (11.3) | 596,013 (11.2) |
| Medium (5–11) | 193,477 (3.6) | 189,894 (3.6) | 186,943 (3.5) | 185,178 (3.5) |
| High (>11) | 103,079 (1.9) | 101,561 (1.9) | 99,978 (1.9) | 99,280 (1.9) |
| Prescription of potentially inadequate medications (number of quarters), n (%) |  |  |  |  |
| None (=0) | 3,669,247 (68.7) | 3,677,219 (68.9) | 3,679,830 (69.1) | 3,677,219 (69.2) |
| Low (1–4) | 1,136,815 (21.3) | 1,133,088 (21.2) | 1,127,348 (21.2) | 1,125,786 (21.2) |
| Medium (5–12) | 282,097 (5.3) | 278,577 (5.2) | 274,794 (5.2) | 271,841 (5.1) |
| High (>12) | 251,699 (4.7) | 246,481 (4.6) | 240,837 (4.5) | 236,083 (4.4) |
| Physiotherapy (number of quarters), n (%) |  |  |  |  |
| None (=0) | 2,559,641 (47.9) | 2,569,405 (48.2) | 2,574,211 (48.4) | 2,568,636 (48.4) |
| Low (1–4) | 2,005,639 (37.6) | 2,000,141 (37.5) | 1,990,238 (37.4) | 1,986,729 (37.4) |
| Medium (5–10) | 578,229 (10.8) | 571,280 (10.7) | 565,361 (10.6) | 563,050 (10.6) |
| High (>10) | 196,349 (3.7) | 194,539 (3.6) | 192,999 (3.6) | 192,514 (3.6) |
| Orthopedic aids prescription, n (%) |  |  |  |  |
| No | 3,080,601 (57.7) | 3,081,584 (57.8) | 3,080,847 (57.9) | 3,072,251 (57.8) |
| Yes | 2,259,257 (42.3) | 2,253,781 (42.2) | 2,241,962 (42.1) | 2,238,678 (42.2) |
| Hearing aids prescription, n (%) |  |  |  |  |
| No | 4,772,025 (89.4) | 4,773,517 (89.5) | 4,767,701 (89.6) | 4,758,263 (89.6) |
| Yes | 567,833 (10.6) | 561,848 (10.5) | 555,108 (10.4) | 552,666 (10.4) |
| Walking aids prescription, n (%) |  |  |  |  |
| No | 4,746,352 (88.9) | 4,756,532 (89.2) | 4,756,691 (89.4) | 4,752,316 (89.5) |
| Yes | 593,506 (11.1) | 578,833 (10.8) | 566,118 (10.6) | 558,613 (10.5) |
| Wheelchairs including mobility scooters prescription, n (%) |  |  |  |  |
| No | 5,282,037 (98.9) | 5,278,679 (98.9) | 5,267,448 (99.0) | 5,256,271 (99.0) |
| Yes | 57,821 (1.1) | 56,686 (1.1) | 55,361 (1.0) | 54,658 (1.0) |
| Aids supporting self-dependence prescription, n (%) |  |  |  |  |
| No | 4,486,135 (84.0) | 4,490,710 (84.2) | 4,488,091 (84.3) | 4,481,618 (84.4) |
| Yes | 853,723 (16.0) | 844,655 (15.8) | 834,718 (15.7) | 829,311 (15.6) |
| Disease-specific aids prescription, n (%) |  |  |  |  |
| No | 3,717,733 (69.6) | 3,711,883 (69.6) | 3,700,550 (69.5) | 3,684,657 (69.4) |
| Yes | 1,622,125 (30.4) | 1,623,482 (30.4) | 1,622,259 (30.5) | 1,626,272 (30.6) |
| **Elixhauser conditions [1]** |  |  |  |  |
| Congestive heart failure, n (%) |  |  |  |  |
| No | 4,991,325 (93.5) | 4,991,269 (93.6) | 4,983,018 (93.6) | 4,968,527 (93.6) |
| Yes | 348,533 (6.5) | 344,096 (6.4) | 339,791 (6.4) | 342,402 (6.4) |
| Cardiac arrythmias, n (%) |  |  |  |  |
| No | 4,788,416 (89.7) | 4,789,319 (89.8) | 4,784,111 (89.9) | 4,765,216 (89.7) |
| Yes | 551,442 (10.3) | 546,046 (10.2) | 538,698 (10.1) | 545,713 (10.3) |
| Renal failure, n (%) |  |  |  |  |
| No | 5,051,772 (94.6) | 5,044,189 (94.5) | 5,028,257 (94.5) | 5,007,495 (94.3) |
| Yes | 288,086 (5.4) | 291,176 (5.5) | 294,552 (5.5) | 303,434 (5.7) |
| Obesity, n (%) |  |  |  |  |
| No | 4,626,890 (86.6) | 4,622,034 (86.6) | 4,612,723 (86.7) | 4,579,078 (86.2) |
| Yes | 712,968 (13.4) | 713,331 (13.4) | 710,086 (13.3) | 731,851 (13.8) |
| Chronic pulmonary disease, n (%) |  |  |  |  |
| No | 4,571,689 (85.6) | 4,580,753 (85.9) | 4,580,839 (86.1) | 4,554,173 (85.8) |
| Yes | 768,169 (14.4) | 754,612 (14.1) | 741,970 (13.9) | 756,756 (14.2) |
| Peptic ulcer disease excluding bleeding, n (%) |  |  |  |  |
| No | 5,298,850 (99.2) | 5,295,272 (99.2) | 5,283,794 (99.3) | 5,271,021 (99.2) |
| Yes | 41,008 (0.8) | 40,093 (0.8) | 39,015 (0.7) | 39,908 (0.8) |
| Weight loss, n (%) |  |  |  |  |
| No | 5,320,634 (99.6) | 5,315,781 (99.6) | 5,302,982 (99.6) | 5,290,732 (99.6) |
| Yes | 19,224 (0.4) | 19,584 (0.4) | 19,827 (0.4) | 20,197 (0.4) |
| Psychoses, n (%) |  |  |  |  |
| No | 5,296,425 (99.2) | 5,292,182 (99.2) | 5,280,242 (99.2) | 5,267,589 (99.2) |
| Yes | 43,433 (0.8) | 43,183 (0.8) | 42,567 (0.8) | 43,340 (0.8) |
| Peripheral valvular disorders, n (%) |  |  |  |  |
| No | 4,912,441 (92.0) | 4,907,224 (92.0) | 4,897,401 (92.0) | 4,876,201 (91.8) |
| Yes | 427,417 (8.0) | 428,141 (8.0) | 425,408 (8.0) | 434,728 (8.2) |
| Metastatic cancer, n (%) |  |  |  |  |
| No | 5,306,872 (99.4) | 5,302,365 (99.4) | 5,289,695 (99.4) | 5,276,818 (99.4) |
| Yes | 32,986 (0.6) | 33,000 (0.6) | 33,114 (0.6) | 34,111 (0.6) |
| Rhematoid arthritis/collagen vascular diseases, n (%) |  |  |  |  |
| No | 5,115,746 (95.8) | 5,113,219 (95.8) | 5,104,520 (95.9) | 5,087,153 (95.8) |
| Yes | 224,112 (4.2) | 222,146 (4.2) | 218,289 (4.1) | 223,776 (4.2) |
| Blood loss anemia, n (%) |  |  |  |  |
| No | 5,332,049 (99.9) | 5,327,510 (99.9) | 5,315,061 (99.9) | 5,302,960 (99.8) |
| Yes | 7,809 (0.1) | 7,855 (0.1) | 7,748 (0.1) | 7,969 (0.2) |
| Deficiency anemia, n (%) |  |  |  |  |
| No | 5,252,345 (98.4) | 5,247,826 (98.4) | 5,235,707 (98.4) | 5,220,947 (98.3) |
| Yes | 87,513 (1.6) | 87,539 (1.6) | 87,102 (1.6) | 89,982 (1.7) |
| Alcohol abuse, n (%) |  |  |  |  |
| No | 5,233,005 (98.0) | 5,228,389 (98.0) | 5,215,174 (98.0) | 5,201,376 (97.9) |
| Yes | 106,853 (2.0) | 106,976 (2.0) | 107,635 (2.0) | 109,553 (2.1) |
| Hypertension, n (%) |  |  |  |  |
| No | 2,384,998 (44.7) | 2,409,496 (45.2) | 2,442,809 (45.9) | 2,384,342 (44.9) |
| Yes, uncomplicated only | 2,600,041 (48.7) | 2,574,894 (48.3) | 2,533,842 (47.6) | 2,576,299 (48.5) |
| Yes, complicated | 354,819 (6.6) | 350,975 (6.6) | 346,158 (6.5) | 350,288 (6.6) |
| Paralysis, n (%) |  |  |  |  |
| No | 5,297,868 (99.2) | 5,293,464 (99.2) | 5,281,176 (99.2) | 5,268,604 (99.2) |
| Yes | 41,990 (0.8) | 41,901 (0.8) | 41,633 (0.8) | 42,325 (0.8) |
| Lymphoma, n (%) |  |  |  |  |
| No | 5,318,229 (99.6) | 5,313,931 (99.6) | 5,301,497 (99.6) | 5,289,187 (99.6) |
| Yes | 21,629 (0.4) | 21,434 (0.4) | 21,312 (0.4) | 21,742 (0.4) |
| Drug abuse, n (%) |  |  |  |  |
| No | 5,316,254 (99.6) | 5,311,514 (99.6) | 5,298,926 (99.6) | 5,286,489 (99.5) |
| Yes | 23,604 (0.4) | 23,851 (0.4) | 23,883 (0.4) | 24,440 (0.5) |
| Depression, n (%) |  |  |  |  |
| No | 4,547,192 (85.2) | 4,544,492 (85.2) | 4,539,372 (85.3) | 4,502,895 (84.8) |
| Yes | 792,666 (14.8) | 790,873 (14.8) | 783,437 (14.7) | 808,034 (15.2) |
| Valvular disease, n (%) |  |  |  |  |
| No | 5,075,426 (95.0) | 5,071,827 (95.1) | 5,062,000 (95.1) | 5,042,933 (95.0) |
| Yes | 264,432 (5.0) | 263,538 (4.9) | 260,809 (4.9) | 267,996 (5.0) |
| Hypothyroidism, n (%) |  |  |  |  |
| No | 4,782,343 (89.6) | 4,776,686 (89.5) | 4,764,398 (89.5) | 4,737,023 (89.2) |
| Yes | 557,515 (10.4) | 558,679 (10.5) | 558,411 (10.5) | 573,906 (10.8) |
| Liver disease, n (%) |  |  |  |  |
| No | 4,851,701 (90.9) | 4,849,920 (90.9) | 4,843,473 (91.0) | 4,818,725 (90.7) |
| Yes | 488,157 (9.1) | 485,445 (9.1) | 479,336 (9.0) | 492,204 (9.3) |
| Fluid and electrolyte disorders, n (%) |  |  |  |  |
| No | 5,221,442 (97.8) | 5,219,192 (97.8) | 5,207,421 (97.8) | 5,196,531 (97.8) |
| Yes | 118,416 (2.2) | 116,173 (2.2) | 115,388 (2.2) | 114,398 (2.2) |
| Other neurological disorders, n (%) |  |  |  |  |
| No | 5,240,548 (98.1) | 5,236,701 (98.2) | 5,225,154 (98.2) | 5,212,002 (98.1) |
| Yes | 99,310 (1.9) | 98,664 (1.8) | 97,655 (1.8) | 98,927 (1.9) |
| Solid tumor without metastasis, n (%) |  |  |  |  |
| No | 4,999,605 (93.6) | 4,997,231 (93.7) | 4,989,721 (93.7) | 4,971,016 (93.6) |
| Yes | 340,253 (6.4) | 338,134 (6.3) | 333,088 (6.3) | 339,913 (6.4) |
| Pulmonary circulation disorders, n (%) |  |  |  |  |
| No | 5,293,591 (99.1) | 5,288,928 (99.1) | 5,276,245 (99.1) | 5,263,762 (99.1) |
| Yes | 46,267 (0.9) | 46,437 (0.9) | 46,564 (0.9) | 47,167 (0.9) |
| Diabetes, n (%) |  |  |  |  |
| No | 3,996,679 (74.8) | 4,000,492 (75.0) | 4,003,327 (75.2) | 3,993,321 (75.2) |
| Yes, uncomplicated only | 930,433 (17.4) | 920,694 (17.3) | 906,575 (17.0) | 893,642 (16.8) |
| Yes, complicated | 412,746 (7.7) | 414,179 (7.8) | 412,907 (7.8) | 423,966 (8.0) |
| Coagulopathy, n (%) |  |  |  |  |
| No | 5,261,400 (98.5) | 5,257,299 (98.5) | 5,245,607 (98.5) | 5,232,941 (98.5) |
| Yes | 78,458 (1.5) | 78,066 (1.5) | 77,202 (1.5) | 77,988 (1.5) |
| **Four conditions as defined by the AOK Research Institute [2]** |  |  |  |  |
| Arthrosis, n (%) |  |  |  |  |
| No | 4,107,433 (76.9) | 4,111,198 (77.1) | 4,119,087 (77.4) | 4,079,651 (76.8) |
| Yes | 1,232,425 (23.1) | 1,224,167 (22.9) | 1,203,722 (22.6) | 1,231,278 (23.2) |
| Chronic obstructive pulmonary disease, n (%) |  |  |  |  |
| No | 4,877,708 (91.3) | 4,879,017 (91.4) | 4,873,856 (91.6) | 4,857,093 (91.5) |
| Yes | 462,150 (8.7) | 456,348 (8.6) | 448,953 (8.4) | 453,836 (8.5) |
| Asthma, n (%) |  |  |  |  |
| No | 5,011,492 (93.9) | 5,006,199 (93.8) | 4,996,666 (93.9) | 4,977,917 (93.7) |
| Yes | 328,366 (6.1) | 329,166 (6.2) | 326,143 (6.1) | 333,012 (6.3) |
| Cogenital heart disease, n (%) |  |  |  |  |
| No | 4,497,484 (84.2) | 4,503,807 (84.4) | 4,505,418 (84.6) | 4,496,706 (84.7) |
| Yes | 842,374 (15.8) | 831,558 (15.6) | 817,391 (15.4) | 814,223 (15.3) |
| **Typical geriatric conditions [3]** |  |  |  |  |
| Decubitus, n (%) |  |  |  |  |
| No | 5,335,836 (99.9) | 5,331,467 (99.9) | 5,319,024 (99.9) | 5,307,308 (99.9) |
| Yes | 4,022 (0.1) | 3,898 (0.1) | 3,785 (0.1) | 3,621 (0.1) |
| Incontinence, n (%) |  |  |  |  |
| No | 5,322,541 (99.7) | 5,318,364 (99.7) | 5,305,743 (99.7) | 5,294,022 (99.7) |
| Yes | 17,317 (0.3) | 17,001 (0.3) | 17,066 (0.3) | 16,907 (0.3) |
| Frailty, n (%) |  |  |  |  |
| No | 5,339,598 (100.0) | 5,335,109 (100.0) | 5,322,565 (100.0) | 5,310,678 (100.0) |
| Yes | 260 (0.0) | 256 (0.0) | 244 (0.0) | 251 (0.0) |
| High risk of complications, n (%) |  |  |  |  |
| No | 5,112,316 (95.7) | 5,110,578 (95.8) | 5,098,050 (95.8) | 5,086,757 (95.8) |
| Yes | 227,542 (4.3) | 224,787 (4.2) | 224,759 (4.2) | 224,172 (4.2) |
| Immobility, n (%) |  |  |  |  |
| No | 5,338,343 (100.0) | 5,333,892 (100.0) | 5,321,362 (100.0) | 5,309,518 (100.0) |
| Yes | 1,515 (0.0) | 1,473 (0.0) | 1,447 (0.0) | 1,411 (0.0) |
| Cognitive deficits, n (%) |  |  |  |  |
| No | 5,328,093 (99.8) | 5,323,808 (99.8) | 5,311,310 (99.8) | 5,299,443 (99.8) |
| Yes | 11,765 (0.2) | 11,557 (0.2) | 11,499 (0.2) | 11,486 (0.2) |
| Medication-associated problems, n (%) |  |  |  |  |
| No | 5,326,195 (99.7) | 5,321,783 (99.7) | 5,309,009 (99.7) | 5,297,024 (99.7) |
| Yes | 13,663 (0.3) | 13,582 (0.3) | 13,800 (0.3) | 13,905 (0.3) |
| Pain, n (%) |  |  |  |  |
| No | 5,258,598 (98.5) | 5,253,216 (98.5) | 5,239,333 (98.4) | 5,226,750 (98.4) |
| Yes | 81,260 (1.5) | 82,149 (1.5) | 83,476 (1.6) | 84,179 (1.6) |
| Sensibility disorders, n (%) |  |  |  |  |
| No | 5,307,388 (99.4) | 5,302,638 (99.4) | 5,289,377 (99.4) | 5,277,331 (99.4) |
| Yes | 32,470 (0.6) | 32,727 (0.6) | 33,432 (0.6) | 33,598 (0.6) |
| Loss of hearing and sight, n (%) |  |  |  |  |
| No | 5,285,492 (99.0) | 5,281,885 (99.0) | 5,269,262 (99.0) | 5,258,304 (99.0) |
| Yes | 54,366 (1.0) | 53,480 (1.0) | 53,547 (1.0) | 52,625 (1.0) |
| Risk of falling and vertigo, n (%) |  |  |  |  |
| No | 5,274,970 (98.8) | 5,270,492 (98.8) | 5,257,063 (98.8) | 5,244,376 (98.7) |
| Yes | 64,888 (1.2) | 64,873 (1.2) | 65,746 (1.2) | 66,553 (1.3) |
| Delayed convalescence, n (%) |  |  |  |  |
| No | 5,339,412 (100.0) | 5,334,896 (100.0) | 5,322,337 (100.0) | 5,310,472 (100.0) |
| Yes | 446 (0.0) | 469 (0.0) | 472 (0.0) | 457 (0.0) |

Note: We did not include the Elixhauser condition for HIV/AIDS. The geriatric conditions depression and anxiety disorder, malnutrition, and fluid and electrolyte disorders were not included, as they were covered by the corresponding Elixhauser conditions psychoses, depression, weight loss, and fluid and electrolyte disorders.

**References**

1. Quan H, Sundararajan V, Halfon P, Fong A, Burnand B, Luthi J-C, et al. Coding Algorithms for Defining Comorbidities in ICD-9-CM and ICD-10 Administrative Data. Medical care. 2005;43(11):1130-9. PMID: 00005650-200511000-00010. doi: 10.1097/01.mlr.0000182534.19832.83.

2. Gesundheitsatlas-Deutschland.de: Methodik. Schüssel K, Schlotmann A, Weirauch H, Brückner G. 2025. URL: <https://www.gesundheitsatlas-deutschland.de/data/Downloads/gesundheitsatlas_deutschland_methodik.pdf> [Accessed 2025-09-04]

3. Lübke N, Meinck M. [Geriatric multimorbidity in claims data - part 1. Analysis of hospital data and long-term care insurance data]. Z Gerontol Geriatr. 2012 Aug;45(6):485-97. PMID: 22538783. doi: 10.1007/s00391-012-0301-y.
