# Supplementary material for: Health Care–Related Determinants of First-Time Long-Term Care Need in Older Adults in Germany: Retrospective Cohort Study Using Claims Data
Source: Interact J Med Res. 2026 Jul 20;15:e86572. doi: 10.2196/86572 (PMC13384046; doi:10.2196/86572)
Supplement: Multimedia Appendix 4 [file ijmr-v15-e86572-s004.docx]

|  | Study population (n=5,339,858) |
| --- | --- |
| Utilization of general practitioner (number of days), n (%) |  |
| None (=0) | 144,622 (2.7) |
| Low (1–38) | 2,608,977 (48.9) |
| Medium (39–79) | 2,099,462 (39.3) |
| High (>79) | 486,797 (9.1) |
| Utilization of specialist, n (%) |  |
| No | 348,302 (6.5) |
| Yes | 4,991,556 (93.5) |
| Utilization of specialist (number of groups), n (%) |  |
| Low (0–4) | 2,764,152 (51.8) |
| Medium (5–7) | 1,760,928 (33.0) |
| High (>7) | 814,778 (15.3) |
| Utilization of specialist (number of days), n (%) |  |
| Low (0–38) | 4,257,604 (79.7) |
| Medium (39–317) | 1,077,686 (20.2) |
| High (>317) | 4,568 (0.1) |
| Hospitalizations, n (%) |  |
| None (=0) | 2,907,688 (54.5) |
| Low (1–2) | 1,782,064 (33.4) |
| Medium (3–5) | 528,055 (9.9) |
| High (>5) | 122,051 (2.3) |
| Screenings and vaccinations (number of services), n (%) |  |
| None (=0) | 978,986 (18.3) |
| Low (=1) | 1,803,274 (33.8) |
| Medium (=2) | 1,434,686 (26.9) |
| High (>2) | 1,122,912 (21.0) |
| Polypharmacy (number of quarters), n (%) |  |
| None (=0) | 4,544,979 (85.1) |
| Low (1–3) | 509,017 (9.5) |
| Medium (4–9) | 191,899 (3.6) |
| High (>9) | 93,963 (1.8) |
| Prescription of potentially inadequate medications (number of quarters), n (%) |  |
| None (=0) | 3,848,620 (72.1) |
| Low (1–3) | 946,474 (17.7) |
| Medium (4–10) | 298,344 (5.6) |
| High (>10) | 246,420 (4.6) |
| Physiotherapy (number of quarters), n (%) |  |
| None (=0) | 2,773,411 (51.9) |
| Low (1–3) | 1,725,746 (32.3) |
| Medium (4–8) | 634,454 (11.9) |
| High (>8) | 206,247 (3.9) |
