# Supplementary material for: Health Care–Related Determinants of First-Time Long-Term Care Need in Older Adults in Germany: Retrospective Cohort Study Using Claims Data
Source: Interact J Med Res. 2026 Jul 20;15:e86572. doi: 10.2196/86572 (PMC13384046; doi:10.2196/86572)
Supplement: Multimedia Appendix 5 [file ijmr-v15-e86572-s005.docx]

7,571,460 persons aged 60 years or older in the first quarter of 2021

658,285 persons excluded who were not continuously insured for reasons other than death or resided abroad during the study period

7,555,903 persons with valid and nonmissing sociodemographic information

15,557 persons excluded who either
- had conflicting information regarding date
 of birth, sex, or date of death (n=5866) or
- had a German zip code that could not be
 mapped to regional variables (n=9698)

5,335,365 persons included in multiple regression analysis

1,562,253 persons excluded who needed long-term care before the start of follow-up

6,897,618 persons observable during the study period
