# Supplementary material for: Health Care–Related Determinants of First-Time Long-Term Care Need in Older Adults in Germany: Retrospective Cohort Study Using Claims Data
Source: Interact J Med Res. 2026 Jul 20;15:e86572. doi: 10.2196/86572 (PMC13384046; doi:10.2196/86572)
Supplement: Multimedia Appendix 8 [file ijmr-v15-e86572-s008.docx]

|  | **Main analysis (n=5,339,858)** | | **Sensitivity analysis 1 (n=5,335,365)** | | **Sensitivity analysis 2 (n=5,322,809)** | | **Sensitivity analysis 3 (n=5,310,929)** | |
| --- | --- | --- | --- | --- | --- | --- | --- | --- |
|  | **Adjusted multiple exposure model** | | | | | | | |
|  | OR (95% CI) | *P* value | OR (95% CI) | *P* value | OR (95% CI) | *P* value | OR (95% CI) | *P* value |
| **Exposure variables** |  |  |  |  |  |  |  |  |
| Utilization of general practitioner (number of days) |  |  |  |  |  |  |  |  |
| None (=0) | Ref. | Ref. | Ref. | Ref. | Ref. | Ref. | Ref. | Ref. |
| Low (1–47) | 1.38 (1.29–1.48) | <.001 | 1.39 (1.29–1.49) | <.001 | 1.22 (1.14–1.31) | <.001 | 1.21 (1.12–1.30) | <.001 |
| Medium (48–98) | 1.59 (1.48–1.71) | <.001 | 1.61 (1.50–1.74) | <.001 | 1.40 (1.30–1.50) | <.001 | 1.34 (1.24–1.45) | <.001 |
| High (>98) | 1.65 (1.53–1.77) | <.001 | 1.68 (1.56–1.81) | <.001 | 1.50 (1.39–1.63) | <.001 | 1.41 (1.31–1.53) | <.001 |
| Utilization of specialist |  |  |  |  |  |  |  |  |
| None (=0 groups and billing days) | Ref. | Ref. | Ref. | Ref. | Ref. | Ref. | Ref. | Ref. |
| Positive number of groups (>0) and billing days (>0) | 0.82 (0.79–0.85) | <.001 | 0.84 (0.81–0.87) | <.001 | 0.84 (0.81–0.87) | <.001 | 0.83 (0.79–0.86) | <.001 |
| Utilization of specialist (number of groups) |  |  |  |  |  |  |  |  |
| Low (0–4) | Ref. | Ref. | Ref. | Ref. | Ref. | Ref. | Ref. | Ref. |
| Medium (5–7) | 0.95 (0.93–0.96) | <.001 | 0.94 (0.92–0.96) | <.001 | 0.91 (0.90–0.93) | <.001 | 0.93 (0.92–0.95) | <.001 |
| High (>7) | 0.94 (0.92–0.96) | <.001 | 0.92 (0.90–0.94) | <.001 | 0.89 (0.87–0.91) | <.001 | 0.91 (0.89–0.94) | <.001 |
| Utilization of specialist (number of days) |  |  |  |  |  |  |  |  |
| Low (0–47) | Ref. | Ref. | Ref. | Ref. | Ref. | Ref. | Ref. | Ref. |
| Medium (48–396) | 1.06 (1.04–1.08) | <.001 | 1.05 (1.03–1.07) | <.001 | 1.10 (1.08–1.13) | <.001 | 1.06 (1.04–1.09) | <.001 |
| High (>396) | 1.70 (1.48–1.95) | <.001 | 1.83 (1.60–2.10) | <.001 | 1.74 (1.51–1.99) | <.001 | 1.90 (1.65–2.18) | <.001 |
| Hospitalizations |  |  |  |  |  |  |  |  |
| None (=0) | Ref. | Ref. | Ref. | Ref. | Ref. | Ref. | Ref. | Ref. |
| Low (1–2) | 1.46 (1.44–1.49) | <.001 | 1.46 (1.44–1.49) | <.001 | 1.51 (1.49–1.54) | <.001 | 1.53 (1.50–1.56) | <.001 |
| Medium (3–6) | 2.00 (1.96–2.05) | <.001 | 2.01 (1.97–2.06) | <.001 | 2.19 (2.14–2.24) | <.001 | 2.19 (2.14–2.24) | <.001 |
| High (>6) | 3.07 (2.97–3.17) | <.001 | 3.10 (2.99–3.20) | <.001 | 3.39 (3.28–3.51) | <.001 | 3.34 (3.23–3.46) | <.001 |
| Screenings and vaccinations (number of services) |  |  |  |  |  |  |  |  |
| None (=0) | Ref. | Ref. | Ref. | Ref. | Ref. | Ref. | Ref. | Ref. |
| Low (=1) | 0.90 (0.88–0.92) | <.001 | 0.90 (0.88–0.92) | <.001 | 0.92 (0.90–0.94) | <.001 | 0.91 (0.89–0.94) | <.001 |
| Medium (=2) | 0.77 (0.76–0.79) | <.001 | 0.78 (0.76–0.80) | <.001 | 0.79 (0.77–0.81) | <.001 | 0.79 (0.77–0.81) | <.001 |
| High (>2) | 0.62 (0.60–0.64) | <.001 | 0.64 (0.63–0.66) | <.001 | 0.65 (0.63–0.66) | <.001 | 0.64 (0.62–0.66) | <.001 |
| DMP congenital heart disease enrolment |  |  |  |  |  |  |  |  |
| No | Ref. | Ref. | Ref. | Ref. | Ref. | Ref. | Ref. | Ref. |
| Yes | 0.93 (0.91–0.96) | <.001 | 0.94 (0.92–0.97) | <.001 | 0.91 (0.88–0.93) | <.001 | 0.91 (0.88–0.93) | <.001 |
| DMP chronic obstructive pulmonary disease enrolment |  |  |  |  |  |  |  |  |
| No | Ref. | Ref. | Ref. | Ref. | Ref. | Ref. | Ref. | Ref. |
| Yes | 1.19 (1.14–1.23) | <.001 | 1.19 (1.15–1.23) | <.001 | 1.23 (1.19–1.28) | <.001 | 1.19 (1.14–1.24) | <.001 |
| DMP asthma enrolment |  |  |  |  |  |  |  |  |
| No | Ref. | Ref. | Ref. | Ref. | Ref. | Ref. | Ref. | Ref. |
| Yes | 0.98 (0.93–1.03) | >0.99 | 0.93 (0.88–0.98) | 0.14 | 0.95 (0.90–1.00) | 0.89 | 0.96 (0.90–1.01) | 0.97 |
| DMP diabetes enrolment |  |  |  |  |  |  |  |  |
| No | Ref. | Ref. | Ref. | Ref. | Ref. | Ref. | Ref. | Ref. |
| Yes | 0.99 (0.96–1.02) | >0.99 | 0.99 (0.96–1.02) | >0.99 | 0.96 (0.93–0.99) | 0.17 | 0.95 (0.92–0.98) | 0.03 |
| Polypharmacy (number of quarters) |  |  |  |  |  |  |  |  |
| None (=0) | Ref. | Ref. | Ref. | Ref. | Ref. | Ref. | Ref. | Ref. |
| Low (1–4) | 1.38 (1.35–1.40) | <.001 | 1.38 (1.36–1.41) | <.001 | 1.37 (1.34–1.39) | <.001 | 1.37 (1.35–1.40) | <.001 |
| Medium (5–11) | 1.49 (1.45–1.53) | <.001 | 1.48 (1.44–1.53) | <.001 | 1.47 (1.43–1.52) | <.001 | 1.50 (1.45–1.54) | <.001 |
| High (>11) | 1.68 (1.62–1.74) | <.001 | 1.59 (1.54–1.65) | <.001 | 1.69 (1.63–1.75) | <.001 | 1.60 (1.54–1.66) | <.001 |
| Prescription of potentially inadequate medications (number of quarters) |  |  |  |  |  |  |  |  |
| None (=0) | Ref. | Ref. | Ref. | Ref. | Ref. | Ref. | Ref. | Ref. |
| Low (1–4) | 1.08 (1.07–1.10) | <.001 | 1.07 (1.05–1.09) | <.001 | 1.08 (1.06–1.10) | <.001 | 1.08 (1.06–1.09) | <.001 |
| Medium (5–12) | 1.11 (1.08–1.14) | <.001 | 1.11 (1.08–1.14) | <.001 | 1.09 (1.06–1.12) | <.001 | 1.11 (1.08–1.14) | <.001 |
| High (>12) | 1.14 (1.11–1.17) | <.001 | 1.18 (1.15–1.21) | <.001 | 1.18 (1.14–1.21) | <.001 | 1.16 (1.13–1.20) | <.001 |
| Physiotherapy (number of quarters) |  |  |  |  |  |  |  |  |
| None (=0) | Ref. | Ref. | Ref. | Ref. | Ref. | Ref. | Ref. | Ref. |
| Low (1–4) | 0.85 (0.84–0.86) | <.001 | 0.86 (0.85–0.88) | <.001 | 0.85 (0.84–0.87) | <.001 | 0.85 (0.83–0.86) | <.001 |
| Medium (5–10) | 0.81 (0.79–0.83) | <.001 | 0.81 (0.79–0.83) | <.001 | 0.80 (0.78–0.82) | <.001 | 0.80 (0.78–0.83) | <.001 |
| High (>10) | 0.98 (0.95–1.01) | >0.99 | 1.00 (0.96–1.03) | >0.99 | 0.94 (0.91–0.97) | 0.02 | 0.94 (0.91–0.98) | 0.03 |
| Orthopedic aids prescription |  |  |  |  |  |  |  |  |
| No | Ref. | Ref. | Ref. | Ref. | Ref. | Ref. | Ref. | Ref. |
| Yes | 0.94 (0.93–0.96) | <.001 | 0.94 (0.93–0.96) | <.001 | 0.93 (0.91–0.94) | <.001 | 0.94 (0.92–0.96) | <.001 |
| Hearing aids prescription |  |  |  |  |  |  |  |  |
| No | Ref. | Ref. | Ref. | Ref. | Ref. | Ref. | Ref. | Ref. |
| Yes | 0.98 (0.96–0.99) | 0.22 | 1.01 (0.99–1.03) | >0.99 | 1.00 (0.98–1.02) | >0.99 | 1.03 (1.01–1.05) | 0.02 |
| Walking aids prescription |  |  |  |  |  |  |  |  |
| No | Ref. | Ref. | Ref. | Ref. | Ref. | Ref. | Ref. | Ref. |
| Yes | 1.62 (1.59–1.64) | <.001 | 1.68 (1.65–1.70) | <.001 | 1.70 (1.67–1.73) | <.001 | 1.69 (1.66–1.72) | <.001 |
| Wheelchairs including mobility scooters prescription |  |  |  |  |  |  |  |  |
| No | Ref. | Ref. | Ref. | Ref. | Ref. | Ref. | Ref. | Ref. |
| Yes | 1.43 (1.38–1.49) | <.001 | 1.46 (1.41–1.52) | <.001 | 1.46 (1.40–1.52) | <.001 | 1.47 (1.41–1.53) | <.001 |
| Aids supporting self-dependence prescription |  |  |  |  |  |  |  |  |
| No | Ref. | Ref. | Ref. | Ref. | Ref. | Ref. | Ref. | Ref. |
| Yes | 1.31 (1.29–1.33) | <.001 | 1.30 (1.28–1.32) | <.001 | 1.27 (1.25–1.29) | <.001 | 1.27 (1.25–1.30) | <.001 |
| Disease-specific aids prescription |  |  |  |  |  |  |  |  |
| No | Ref. | Ref. | Ref. | Ref. | Ref. | Ref. | Ref. | Ref. |
| Yes | 1.14 (1.12–1.16) | <.001 | 1.14 (1.12–1.16) | <.001 | 1.13 (1.11–1.15) | <.001 | 1.14 (1.12–1.16) | <.001 |
| **Covariates** |  |  |  |  |  |  |  |  |
| Age in years | 1.14 (1.14–1.14) | <.001 | 1.13 (1.13–1.13) | <.001 | 1.12 (1.12–1.13) | <.001 | 1.12 (1.12–1.12) | <.001 |
| Sex |  |  |  |  |  |  |  |  |
| Female | 0.93 (0.92–0.95) | <.001 | 0.91 (0.90–0.93) | <.001 | 0.91 (0.90–0.93) | <.001 | 0.92 (0.91–0.94) | <.001 |
| Male | Ref. | Ref. | Ref. | Ref. | Ref. | Ref. | Ref. | Ref. |
| German Index of Social Deprivation | 2.27 (2.17–2.38) | <.001 | 2.26 (2.16–2.37) | <.001 | 2.34 (2.23–2.46) | <.001 | 2.20 (2.09–2.31) | <.001 |
| County settlement structure type |  |  |  |  |  |  |  |  |
| Metropolitan | Ref. | Ref. | Ref. | Ref. | Ref. | Ref. | Ref. | Ref. |
| Urban | 1.01 (1.00–1.03) | 0.13 | 1.01 (1.00–1.03) | 0.14 | 1.01 (0.99–1.03) | 0.18 | 0.96 (0.94–0.98) | <.001 |
| Rural with agglomeration tendency | 0.89 (0.87–0.90) | <.001 | 0.87 (0.86–0.89) | <.001 | 0.91 (0.89–0.93) | <.001 | 0.86 (0.84–0.88) | <.001 |
| Rural | 0.86 (0.84–0.88) | <.001 | 0.86 (0.84–0.88) | <.001 | 0.88 (0.86–0.90) | <.001 | 0.86 (0.84–0.88) | <.001 |
| **Elixhauser conditions** |  |  |  |  |  |  |  |  |
| Congestive heart failure |  |  |  |  |  |  |  |  |
| No | Ref. | Ref. | Ref. | Ref. | Ref. | Ref. | Ref. | Ref. |
| Yes | 1.04 (1.02–1.06) | <.001 | 1.07 (1.05–1.10) | <.001 | 1.04 (1.02–1.07) | <.001 | 1.07 (1.04–1.09) | <.001 |
| Cardiac arrythmias |  |  |  |  |  |  |  |  |
| No | Ref. | Ref. | Ref. | Ref. | Ref. | Ref. | Ref. | Ref. |
| Yes | 0.98 (0.96–1.00) | 0.06 | 0.98 (0.96–1.00) | 0.03 | 0.97 (0.95–0.99) | 0.01 | 0.98 (0.96–1.00) | 0.03 |
| Renal failure |  |  |  |  |  |  |  |  |
| No | Ref. | Ref. | Ref. | Ref. | Ref. | Ref. | Ref. | Ref. |
| Yes | 0.97 (0.95–0.99) | 0.009 | 0.99 (0.97–1.02) | 0.58 | 0.99 (0.97–1.02) | 0.53 | 0.98 (0.96–1.01) | 0.17 |
| Obesity |  |  |  |  |  |  |  |  |
| No | Ref. | Ref. | Ref. | Ref. | Ref. | Ref. | Ref. | Ref. |
| Yes | 1.21 (1.19–1.23) | <.001 | 1.18 (1.16–1.21) | <.001 | 1.17 (1.15–1.19) | <.001 | 1.14 (1.12–1.16) | <.001 |
| Chronic pulmonary disease |  |  |  |  |  |  |  |  |
| No | Ref. | Ref. | Ref. | Ref. | Ref. | Ref. | Ref. | Ref. |
| Yes | 1.08 (1.04–1.13) | <.001 | 1.09 (1.05–1.13) | <.001 | 1.06 (1.02–1.11) | 0.004 | 1.06 (1.02–1.10) | 0.007 |
| Peptic ulcer disease excluding bleeding |  |  |  |  |  |  |  |  |
| No | Ref. | Ref. | Ref. | Ref. | Ref. | Ref. | Ref. | Ref. |
| Yes | 0.98 (0.92–1.05) | 0.60 | 1.07 (1.01–1.15) | 0.03 | 0.99 (0.92–1.06) | 0.73 | 1.01 (0.94–1.08) | 0.79 |
| Weight loss |  |  |  |  |  |  |  |  |
| No | Ref. | Ref. | Ref. | Ref. | Ref. | Ref. | Ref. | Ref. |
| Yes | 1.18 (1.09–1.29) | <.001 | 1.13 (1.03–1.23) | 0.006 | 1.26 (1.16–1.36) | <.001 | 1.16 (1.06–1.26) | <.001 |
| Psychoses |  |  |  |  |  |  |  |  |
| No | Ref. | Ref. | Ref. | Ref. | Ref. | Ref. | Ref. | Ref. |
| Yes | 1.97 (1.85–2.10) | <.001 | 1.83 (1.72–1.96) | <.001 | 1.89 (1.77–2.02) | <.001 | 1.90 (1.78–2.03) | <.001 |
| Peripheral valvular disorders |  |  |  |  |  |  |  |  |
| No | Ref. | Ref. | Ref. | Ref. | Ref. | Ref. | Ref. | Ref. |
| Yes | 1.01 (0.99–1.03) | 0.31 | 1.01 (0.99–1.03) | 0.22 | 1.04 (1.02–1.06) | <.001 | 1.04 (1.02–1.07) | <.001 |
| Metastatic cancer |  |  |  |  |  |  |  |  |
| No | Ref. | Ref. | Ref. | Ref. | Ref. | Ref. | Ref. | Ref. |
| Yes | 1.20 (1.12–1.28) | <.001 | 1.21 (1.13–1.29) | <.001 | 1.25 (1.17–1.34) | <.001 | 1.25 (1.17–1.33) | <.001 |
| Rhematoid arthritis/collagen vascular diseases |  |  |  |  |  |  |  |  |
| No | Ref. | Ref. | Ref. | Ref. | Ref. | Ref. | Ref. | Ref. |
| Yes | 1.05 (1.02–1.08) | <.001 | 1.04 (1.01–1.07) | 0.01 | 1.06 (1.03–1.09) | <.001 | 1.03 (1.00–1.06) | 0.04 |
| Blood loss anemia |  |  |  |  |  |  |  |  |
| No | Ref. | Ref. | Ref. | Ref. | Ref. | Ref. | Ref. | Ref. |
| Yes | 0.97 (0.84–1.10) | 0.61 | 0.99 (0.86–1.13) | 0.89 | 1.01 (0.88–1.16) | 0.91 | 1.13 (0.99–1.30) | 0.07 |
| Deficiency anemia |  |  |  |  |  |  |  |  |
| No | Ref. | Ref. | Ref. | Ref. | Ref. | Ref. | Ref. | Ref. |
| Yes | 1.07 (1.03–1.12) | 0.001 | 0.98 (0.94–1.03) | 0.40 | 1.08 (1.03–1.13) | <.001 | 1.02 (0.98–1.07) | 0.29 |
| Alcohol abuse |  |  |  |  |  |  |  |  |
| No | Ref. | Ref. | Ref. | Ref. | Ref. | Ref. | Ref. | Ref. |
| Yes | 1.86 (1.78–1.95) | <.001 | 1.76 (1.68–1.84) | <.001 | 1.79 (1.72–1.88) | <.001 | 1.69 (1.62–1.77) | <.001 |
| Hypertension |  |  |  |  |  |  |  |  |
| No | Ref. | Ref. | Ref. | Ref. | Ref. | Ref. | Ref. | Ref. |
| Yes, uncomplicated only | 0.94 (0.93–0.96) | <.001 | 0.98 (0.96–0.99) | 0.009 | 1.00 (0.98–1.02) | 0.92 | 1.00 (0.98–1.01) | 0.63 |
| Yes, complicated | 0.91 (0.89–0.94) | <.001 | 0.93 (0.91–0.96) | <.001 | 0.95 (0.92–0.98) | <.001 | 0.97 (0.94–1.00) | 0.03 |
| Paralysis |  |  |  |  |  |  |  |  |
| No | Ref. | Ref. | Ref. | Ref. | Ref. | Ref. | Ref. | Ref. |
| Yes | 1.41 (1.34–1.49) | <.001 | 1.40 (1.33–1.48) | <.001 | 1.39 (1.31–1.47) | <.001 | 1.32 (1.24–1.40) | <.001 |
| Lymphoma |  |  |  |  |  |  |  |  |
| No | Ref. | Ref. | Ref. | Ref. | Ref. | Ref. | Ref. | Ref. |
| Yes | 1.16 (1.07–1.26) | <.001 | 1.18 (1.08–1.28) | <.001 | 1.19 (1.10–1.30) | <.001 | 1.12 (1.02–1.22) | 0.01 |
| Drug abuse |  |  |  |  |  |  |  |  |
| No | Ref. | Ref. | Ref. | Ref. | Ref. | Ref. | Ref. | Ref. |
| Yes | 1.15 (1.06–1.25) | <.001 | 1.16 (1.07–1.27) | <.001 | 1.15 (1.05–1.25) | <.001 | 1.18 (1.08–1.29) | <.001 |
| Depression |  |  |  |  |  |  |  |  |
| No | Ref. | Ref. | Ref. | Ref. | Ref. | Ref. | Ref. | Ref. |
| Yes | 1.13 (1.11–1.16) | <.001 | 1.12 (1.10–1.14) | <.001 | 1.11 (1.09–1.13) | <.001 | 1.14 (1.12–1.16) | <.001 |
| Valvular disease |  |  |  |  |  |  |  |  |
| No | Ref. | Ref. | Ref. | Ref. | Ref. | Ref. | Ref. | Ref. |
| Yes | 0.97 (0.94–0.99) | 0.01 | 0.96 (0.94–0.99) | 0.002 | 1.00 (0.97–1.02) | 0.82 | 0.97 (0.95–1.00) | 0.03 |
| Hypothyroidism |  |  |  |  |  |  |  |  |
| No | Ref. | Ref. | Ref. | Ref. | Ref. | Ref. | Ref. | Ref. |
| Yes | 0.94 (0.92–0.96) | <.001 | 0.93 (0.91–0.95) | <.001 | 0.94 (0.91–0.96) | <.001 | 0.93 (0.91–0.95) | <.001 |
| Liver disease |  |  |  |  |  |  |  |  |
| No | Ref. | Ref. | Ref. | Ref. | Ref. | Ref. | Ref. | Ref. |
| Yes | 0.99 (0.96–1.01) | 0.19 | 1.00 (0.98–1.02) | 0.82 | 0.97 (0.95–1.00) | 0.02 | 1.00 (0.98–1.02) | 0.85 |
| Fluid and electrolyte disorders |  |  |  |  |  |  |  |  |
| No | Ref. | Ref. | Ref. | Ref. | Ref. | Ref. | Ref. | Ref. |
| Yes | 1.01 (0.98–1.05) | 0.41 | 1.05 (1.02–1.09) | 0.004 | 1.04 (1.00–1.08) | 0.03 | 1.02 (0.98–1.06) | 0.33 |
| Other neurological disorders |  |  |  |  |  |  |  |  |
| No | Ref. | Ref. | Ref. | Ref. | Ref. | Ref. | Ref. | Ref. |
| Yes | 1.40 (1.35–1.45) | <.001 | 1.40 (1.35–1.46) | <.001 | 1.40 (1.35–1.46) | <.001 | 1.43 (1.37–1.48) | <.001 |
| Solid tumor without metastasis |  |  |  |  |  |  |  |  |
| No | Ref. | Ref. | Ref. | Ref. | Ref. | Ref. | Ref. | Ref. |
| Yes | 1.03 (1.00–1.05) | 0.03 | 1.06 (1.04–1.09) | <.001 | 1.08 (1.05–1.10) | <.001 | 1.06 (1.03–1.09) | <.001 |
| Pulmonary circulation disorders |  |  |  |  |  |  |  |  |
| No | Ref. | Ref. | Ref. | Ref. | Ref. | Ref. | Ref. | Ref. |
| Yes | 1.03 (0.98–1.09) | 0.18 | 1.01 (0.96–1.06) | 0.79 | 1.00 (0.95–1.05) | 0.95 | 1.04 (0.99–1.10) | 0.11 |
| Diabetes |  |  |  |  |  |  |  |  |
| No | Ref. | Ref. | Ref. | Ref. | Ref. | Ref. | Ref. | Ref. |
| Yes, uncomplicated only | 1.11 (1.08–1.14) | <.001 | 1.08 (1.05–1.11) | <.001 | 1.09 (1.06–1.13) | <.001 | 1.12 (1.09–1.16) | <.001 |
| Yes, complicated | 1.12 (1.09–1.16) | <.001 | 1.08 (1.05–1.12) | <.001 | 1.12 (1.08–1.16) | <.001 | 1.14 (1.10–1.18) | <.001 |
| Coagulopathy |  |  |  |  |  |  |  |  |
| No | Ref. | Ref. | Ref. | Ref. | Ref. | Ref. | Ref. | Ref. |
| Yes | 0.98 (0.94–1.02) | 0.39 | 1.04 (1.00–1.09) | 0.06 | 1.02 (0.98–1.07) | 0.40 | 1.04 (0.99–1.09) | 0.10 |
| **Four conditions as defined by the AOK Research Institute** |  |  |  |  |  |  |  |  |
| Arthrosis |  |  |  |  |  |  |  |  |
| No | Ref. | Ref. | Ref. | Ref. | Ref. | Ref. | Ref. | Ref. |
| Yes | 1.01 (0.99–1.02) | 0.46 | 1.03 (1.01–1.04) | <.001 | 1.04 (1.02–1.05) | <.001 | 1.04 (1.02–1.05) | <.001 |
| Chronic obstructive pulmonary disease |  |  |  |  |  |  |  |  |
| No | Ref. | Ref. | Ref. | Ref. | Ref. | Ref. | Ref. | Ref. |
| Yes | 1.12 (1.07–1.17) | <.001 | 1.12 (1.08–1.17) | <.001 | 1.17 (1.12–1.22) | <.001 | 1.15 (1.10–1.20) | <.001 |
| Asthma |  |  |  |  |  |  |  |  |
| No | Ref. | Ref. | Ref. | Ref. | Ref. | Ref. | Ref. | Ref. |
| Yes | 0.93 (0.90–0.97) | 0.001 | 0.93 (0.89–0.97) | <.001 | 0.94 (0.90–0.98) | 0.008 | 0.93 (0.89–0.97) | <.001 |
| Cogenital heart disease |  |  |  |  |  |  |  |  |
| No | Ref. | Ref. | Ref. | Ref. | Ref. | Ref. | Ref. | Ref. |
| Yes | 0.96 (0.94–0.98) | <.001 | 0.95 (0.93–0.97) | <.001 | 0.98 (0.96–1.00) | 0.09 | 1.00 (0.97–1.02) | 0.80 |
| **Typical geriatric conditions** |  |  |  |  |  |  |  |  |
| Decubitus |  |  |  |  |  |  |  |  |
| No | Ref. | Ref. | Ref. | Ref. | Ref. | Ref. | Ref. | Ref. |
| Yes | 1.28 (1.09–1.49) | 0.002 | 1.39 (1.18–1.62) | <.001 | 1.34 (1.14–1.58) | <.001 | 1.29 (1.08–1.54) | 0.004 |
| Incontinence |  |  |  |  |  |  |  |  |
| No | Ref. | Ref. | Ref. | Ref. | Ref. | Ref. | Ref. | Ref. |
| Yes | 1.04 (0.96–1.13) | 0.29 | 1.01 (0.93–1.09) | 0.90 | 1.01 (0.93–1.10) | 0.80 | 1.04 (0.96–1.14) | 0.33 |
| Frailty |  |  |  |  |  |  |  |  |
| No | Ref. | Ref. | Ref. | Ref. | Ref. | Ref. | Ref. | Ref. |
| Yes | 1.22 (0.79–1.90) | 0.37 | 1.10 (0.68–1.78) | 0.70 | 1.14 (0.69–1.88) | 0.61 | 0.79 (0.42–1.46) | 0.44 |
| High risk of complications |  |  |  |  |  |  |  |  |
| No | Ref. | Ref. | Ref. | Ref. | Ref. | Ref. | Ref. | Ref. |
| Yes | 1.02 (1.00–1.05) | 0.09 | 1.01 (0.98–1.04) | 0.53 | 0.98 (0.96–1.01) | 0.27 | 1.00 (0.97–1.03) | 0.83 |
| Immobility |  |  |  |  |  |  |  |  |
| No | Ref. | Ref. | Ref. | Ref. | Ref. | Ref. | Ref. | Ref. |
| Yes | 1.16 (0.90–1.51) | 0.25 | 1.02 (0.77–1.34) | 0.91 | 1.02 (0.76–1.35) | 0.91 | 0.92 (0.68–1.25) | 0.58 |
| Cognitive deficits |  |  |  |  |  |  |  |  |
| No | Ref. | Ref. | Ref. | Ref. | Ref. | Ref. | Ref. | Ref. |
| Yes | 1.36 (1.25–1.47) | <.001 | 1.23 (1.13–1.35) | <.001 | 1.34 (1.23–1.46) | <.001 | 1.45 (1.33–1.58) | <.001 |
| Medication-associated problems |  |  |  |  |  |  |  |  |
| No | Ref. | Ref. | Ref. | Ref. | Ref. | Ref. | Ref. | Ref. |
| Yes | 0.91 (0.83–1.01) | 0.07 | 0.93 (0.84–1.02) | 0.13 | 0.93 (0.84–1.03) | 0.16 | 0.93 (0.84–1.03) | 0.19 |
| Pain |  |  |  |  |  |  |  |  |
| No | Ref. | Ref. | Ref. | Ref. | Ref. | Ref. | Ref. | Ref. |
| Yes | 1.02 (0.98–1.07) | 0.36 | 1.01 (0.97–1.06) | 0.55 | 1.00 (0.96–1.05) | 0.90 | 1.03 (0.98–1.08) | 0.22 |
| Sensibility disorders |  |  |  |  |  |  |  |  |
| No | Ref. | Ref. | Ref. | Ref. | Ref. | Ref. | Ref. | Ref. |
| Yes | 1.07 (1.00–1.14) | 0.04 | 1.05 (0.99–1.12) | 0.13 | 1.01 (0.95–1.08) | 0.77 | 1.10 (1.03–1.17) | 0.005 |
| Loss of hearing and sight |  |  |  |  |  |  |  |  |
| No | Ref. | Ref. | Ref. | Ref. | Ref. | Ref. | Ref. | Ref. |
| Yes | 1.01 (0.96–1.06) | 0.84 | 0.97 (0.92–1.02) | 0.27 | 0.95 (0.90–1.01) | 0.08 | 0.96 (0.91–1.02) | 0.16 |
| Risk of falling and vertigo |  |  |  |  |  |  |  |  |
| No | Ref. | Ref. | Ref. | Ref. | Ref. | Ref. | Ref. | Ref. |
| Yes | 1.00 (0.95–1.04) | 0.89 | 1.00 (0.95–1.04) | 0.84 | 1.03 (0.98–1.08) | 0.24 | 0.97 (0.93–1.02) | 0.28 |
| Delayed convalescence |  |  |  |  |  |  |  |  |
| No | Ref. | Ref. | Ref. | Ref. | Ref. | Ref. | Ref. | Ref. |
| Yes | 0.77 (0.50–1.20) | 0.25 | 0.88 (0.57–1.35) | 0.55 | 1.20 (0.81–1.77) | 0.36 | 1.11 (0.73–1.69) | 0.62 |
|  |  |  |  |  |  |  |  |  |
|  |  |  |  |  |  |  |  |  |
