# Supplementary material for: Health Care–Related Determinants of First-Time Long-Term Care Need in Older Adults in Germany: Retrospective Cohort Study Using Claims Data
Source: Interact J Med Res. 2026 Jul 20;15:e86572. doi: 10.2196/86572 (PMC13384046; doi:10.2196/86572)
Supplement: Multimedia Appendix 9 [file ijmr-v15-e86572-s009.docx]

|  | **Main analysis (n=5,339,858)** | | **Sensitivity analysis 4 (n=5,339,858)** | |
| --- | --- | --- | --- | --- |
|  | **Adjusted multiple exposure model** | | | |
|  | OR (95% CI) | *P* value | OR (95% CI) | *P* value |
| **Exposure variables** |  |  |  |  |
| Utilization of general practitioner (number of days) |  |  |  |  |
| None (=0) | Ref. | Ref. | Ref. | Ref. |
| Low (1–47) | 1.38 (1.29–1.48) | <.001 | 1.24 (1.20–1.28) | <.001 |
| Medium (48–98) | 1.59 (1.48–1.71) | <.001 | 1.49 (1.44–1.54) | <.001 |
| High (>98) | 1.65 (1.53–1.77) | <.001 | 1.60 (1.54–1.67) | <.001 |
| Utilization of specialist |  |  |  |  |
| None (=0 groups and billing days) | Ref. | Ref. | Ref. | Ref. |
| Positive number of groups (>0) and billing days (>0) | 0.82 (0.79–0.85) | <.001 | 0.84 (0.82–0.86) | <.001 |
| Utilization of specialist (number of groups) |  |  |  |  |
| Low (0–4) | Ref. | Ref. | Ref. | Ref. |
| Medium (5–7) | 0.95 (0.93–0.96) | <.001 | 0.93 (0.92–0.94) | <.001 |
| High (>7) | 0.94 (0.92–0.96) | <.001 | 0.91 (0.89–0.92) | <.001 |
| Utilization of specialist (number of days) |  |  |  |  |
| Low (0–47) | Ref. | Ref. | Ref. | Ref. |
| Medium (48–396) | 1.06 (1.04–1.08) | <.001 | 1.09 (1.08–1.11) | <.001 |
| High (>396) | 1.70 (1.48–1.95) | <.001 | 2.04 (1.88–2.21) | <.001 |
| Hospitalizations |  |  |  |  |
| None (=0) | Ref. | Ref. | Ref. | Ref. |
| Low (1–2) | 1.46 (1.44–1.49) | <.001 | 1.27 (1.26–1.29) | <.001 |
| Medium (3–6) | 2.00 (1.96–2.05) | <.001 | 1.65 (1.63–1.67) | <.001 |
| High (>6) | 3.07 (2.97–3.17) | <.001 | 2.41 (2.36–2.46) | <.001 |
| Screenings and vaccinations (number of services) |  |  |  |  |
| None (=0) | Ref. | Ref. | Ref. | Ref. |
| Low (=1) | 0.90 (0.88–0.92) | <.001 | 0.90 (0.89–0.91) | <.001 |
| Medium (=2) | 0.77 (0.76–0.79) | <.001 | 0.76 (0.75–0.77) | <.001 |
| High (>2) | 0.62 (0.60–0.64) | <.001 | 0.61 (0.60–0.62) | <.001 |
| DMP congenital heart disease enrolment |  |  |  |  |
| No | Ref. | Ref. | Ref. | Ref. |
| Yes | 0.93 (0.91–0.96) | <.001 | 0.92 (0.90–0.93) | <.001 |
| DMP chronic obstructive pulmonary disease enrolment |  |  |  |  |
| No | Ref. | Ref. | Ref. | Ref. |
| Yes | 1.19 (1.14–1.23) | <.001 | 1.22 (1.19–1.24) | <.001 |
| DMP asthma enrolment |  |  |  |  |
| No | Ref. | Ref. | Ref. | Ref. |
| Yes | 0.98 (0.93–1.03) | >0.99 | 0.95 (0.92–0.98) | 0.008 |
| DMP diabetes enrolment |  |  |  |  |
| No | Ref. | Ref. | Ref. | Ref. |
| Yes | 0.99 (0.96–1.02) | >0.99 | 0.95 (0.93–0.96) | <.001 |
| Polypharmacy (number of quarters) |  |  |  |  |
| None (=0) | Ref. | Ref. | Ref. | Ref. |
| Low (1–4) | 1.38 (1.35–1.40) | <.001 | 1.37 (1.36–1.39) | <.001 |
| Medium (5–11) | 1.49 (1.45–1.53) | <.001 | 1.55 (1.52–1.57) | <.001 |
| High (>11) | 1.68 (1.62–1.74) | <.001 | 1.74 (1.71–1.78) | <.001 |
| Prescription of potentially inadequate medications (number of quarters) |  |  |  |  |
| None (=0) | Ref. | Ref. | Ref. | Ref. |
| Low (1–4) | 1.08 (1.07–1.10) | <.001 | 1.07 (1.06–1.08) | <.001 |
| Medium (5–12) | 1.11 (1.08–1.14) | <.001 | 1.13 (1.11–1.15) | <.001 |
| High (>12) | 1.14 (1.11–1.17) | <.001 | 1.19 (1.17–1.21) | <.001 |
| Physiotherapy (number of quarters) |  |  |  |  |
| None (=0) | Ref. | Ref. | Ref. | Ref. |
| Low (1–4) | 0.85 (0.84–0.86) | <.001 | 0.86 (0.85–0.87) | <.001 |
| Medium (5–10) | 0.81 (0.79–0.83) | <.001 | 0.83 (0.82–0.84) | <.001 |
| High (>10) | 0.98 (0.95–1.01) | >0.99 | 1.00 (0.98–1.02) | >0.99 |
| Orthopedic aids prescription |  |  |  |  |
| No | Ref. | Ref. | Ref. | Ref. |
| Yes | 0.94 (0.93–0.96) | <.001 | 0.93 (0.92–0.94) | <.001 |
| Hearing aids prescription |  |  |  |  |
| No | Ref. | Ref. | Ref. | Ref. |
| Yes | 0.98 (0.96–0.99) | 0.22 | 1.05 (1.04–1.06) | <.001 |
| Walking aids prescription |  |  |  |  |
| No | Ref. | Ref. | Ref. | Ref. |
| Yes | 1.62 (1.59–1.64) | <.001 | 1.68 (1.66–1.70) | <.001 |
| Wheelchairs including mobility scooters prescription |  |  |  |  |
| No | Ref. | Ref. | Ref. | Ref. |
| Yes | 1.43 (1.38–1.49) | <.001 | 1.37 (1.34–1.40) | <.001 |
| Aids supporting self-dependence prescription |  |  |  |  |
| No | Ref. | Ref. | Ref. | Ref. |
| Yes | 1.31 (1.29–1.33) | <.001 | 1.30 (1.29–1.32) | <.001 |
| Disease-specific aids prescription |  |  |  |  |
| No | Ref. | Ref. | Ref. | Ref. |
| Yes | 1.14 (1.12–1.16) | <.001 | 1.12 (1.12–1.13) | <.001 |
| **Covariates** |  |  |  |  |
| Age in years | 1.14 (1.14–1.14) | <.001 | 1.13 (1.13–1.13) | <.001 |
| Sex |  |  |  |  |
| Female | 0.93 (0.92–0.95) | <.001 | 0.91 (0.90–0.91) | <.001 |
| Male | Ref. | Ref. | Ref. | Ref. |
| German Index of Social Deprivation | 2.27 (2.17–2.38) | <.001 | 2.40 (2.34–2.46) | <.001 |
| County settlement structure type |  |  |  |  |
| Metropolitan | Ref. | Ref. | Ref. | Ref. |
| Urban | 1.01 (1.00–1.03) | 0.13 | 1.00 (0.99–1.01) | 0.50 |
| Rural with agglomeration tendency | 0.89 (0.87–0.90) | <.001 | 0.89 (0.88–0.90) | <.001 |
| Rural | 0.86 (0.84–0.88) | <.001 | 0.85 (0.84–0.86) | <.001 |
| **Elixhauser conditions** |  |  |  |  |
| Congestive heart failure |  |  |  |  |
| No | Ref. | Ref. | Ref. | Ref. |
| Yes | 1.04 (1.02–1.06) | <.001 | 1.08 (1.07–1.09) | <.001 |
| Cardiac arrythmias |  |  |  |  |
| No | Ref. | Ref. | Ref. | Ref. |
| Yes | 0.98 (0.96–1.00) | 0.06 | 1.00 (0.99–1.01) | 0.68 |
| Renal failure |  |  |  |  |
| No | Ref. | Ref. | Ref. | Ref. |
| Yes | 0.97 (0.95–0.99) | 0.009 | 1.01 (1.00–1.03) | 0.04 |
| Obesity |  |  |  |  |
| No | Ref. | Ref. | Ref. | Ref. |
| Yes | 1.21 (1.19–1.23) | <.001 | 1.17 (1.15–1.18) | <.001 |
| Chronic pulmonary disease |  |  |  |  |
| No | Ref. | Ref. | Ref. | Ref. |
| Yes | 1.08 (1.04–1.13) | <.001 | 1.09 (1.07–1.11) | <.001 |
| Peptic ulcer disease excluding bleeding |  |  |  |  |
| No | Ref. | Ref. | Ref. | Ref. |
| Yes | 0.98 (0.92–1.05) | 0.60 | 1.00 (0.97–1.04) | 0.94 |
| Weight loss |  |  |  |  |
| No | Ref. | Ref. | Ref. | Ref. |
| Yes | 1.18 (1.09–1.29) | <.001 | 1.20 (1.14–1.26) | <.001 |
| Psychoses |  |  |  |  |
| No | Ref. | Ref. | Ref. | Ref. |
| Yes | 1.97 (1.85–2.10) | <.001 | 1.94 (1.88–2.02) | <.001 |
| Peripheral valvular disorders |  |  |  |  |
| No | Ref. | Ref. | Ref. | Ref. |
| Yes | 1.01 (0.99–1.03) | 0.31 | 1.05 (1.04–1.07) | <.001 |
| Metastatic cancer |  |  |  |  |
| No | Ref. | Ref. | Ref. | Ref. |
| Yes | 1.20 (1.12–1.28) | <.001 | 1.23 (1.19–1.28) | <.001 |
| Rhematoid arthritis/collagen vascular diseases |  |  |  |  |
| No | Ref. | Ref. | Ref. | Ref. |
| Yes | 1.05 (1.02–1.08) | <.001 | 1.06 (1.05–1.08) | <.001 |
| Blood loss anemia |  |  |  |  |
| No | Ref. | Ref. | Ref. | Ref. |
| Yes | 0.97 (0.84–1.10) | 0.61 | 1.00 (0.93–1.09) | 0.92 |
| Deficiency anemia |  |  |  |  |
| No | Ref. | Ref. | Ref. | Ref. |
| Yes | 1.07 (1.03–1.12) | 0.001 | 1.06 (1.03–1.08) | <.001 |
| Alcohol abuse |  |  |  |  |
| No | Ref. | Ref. | Ref. | Ref. |
| Yes | 1.86 (1.78–1.95) | <.001 | 1.80 (1.76–1.84) | <.001 |
| Hypertension |  |  |  |  |
| No | Ref. | Ref. | Ref. | Ref. |
| Yes, uncomplicated only | 0.94 (0.93–0.96) | <.001 | 0.99 (0.98–1.00) | 0.23 |
| Yes, complicated | 0.91 (0.89–0.94) | <.001 | 0.95 (0.94–0.97) | <.001 |
| Paralysis |  |  |  |  |
| No | Ref. | Ref. | Ref. | Ref. |
| Yes | 1.41 (1.34–1.49) | <.001 | 1.45 (1.41–1.50) | <.001 |
| Lymphoma |  |  |  |  |
| No | Ref. | Ref. | Ref. | Ref. |
| Yes | 1.16 (1.07–1.26) | <.001 | 1.18 (1.13–1.24) | <.001 |
| Drug abuse |  |  |  |  |
| No | Ref. | Ref. | Ref. | Ref. |
| Yes | 1.15 (1.06–1.25) | <.001 | 1.16 (1.11–1.22) | <.001 |
| Depression |  |  |  |  |
| No | Ref. | Ref. | Ref. | Ref. |
| Yes | 1.13 (1.11–1.16) | <.001 | 1.13 (1.11–1.14) | <.001 |
| Valvular disease |  |  |  |  |
| No | Ref. | Ref. | Ref. | Ref. |
| Yes | 0.97 (0.94–0.99) | 0.01 | 0.99 (0.98–1.00) | 0.13 |
| Hypothyroidism |  |  |  |  |
| No | Ref. | Ref. | Ref. | Ref. |
| Yes | 0.94 (0.92–0.96) | <.001 | 0.93 (0.92–0.94) | <.001 |
| Liver disease |  |  |  |  |
| No | Ref. | Ref. | Ref. | Ref. |
| Yes | 0.99 (0.96–1.01) | 0.19 | 0.99 (0.97–1.00) | 0.02 |
| Fluid and electrolyte disorders |  |  |  |  |
| No | Ref. | Ref. | Ref. | Ref. |
| Yes | 1.01 (0.98–1.05) | 0.41 | 1.06 (1.04–1.08) | <.001 |
| Other neurological disorders |  |  |  |  |
| No | Ref. | Ref. | Ref. | Ref. |
| Yes | 1.40 (1.35–1.45) | <.001 | 1.47 (1.44–1.50) | <.001 |
| Solid tumor without metastasis |  |  |  |  |
| No | Ref. | Ref. | Ref. | Ref. |
| Yes | 1.03 (1.00–1.05) | 0.03 | 1.09 (1.08–1.11) | <.001 |
| Pulmonary circulation disorders |  |  |  |  |
| No | Ref. | Ref. | Ref. | Ref. |
| Yes | 1.03 (0.98–1.09) | 0.18 | 1.03 (1.00–1.06) | 0.03 |
| Diabetes |  |  |  |  |
| No | Ref. | Ref. | Ref. | Ref. |
| Yes, uncomplicated only | 1.11 (1.08–1.14) | <.001 | 1.13 (1.11–1.14) | <.001 |
| Yes, complicated | 1.12 (1.09–1.16) | <.001 | 1.15 (1.13–1.17) | <.001 |
| Coagulopathy |  |  |  |  |
| No | Ref. | Ref. | Ref. | Ref. |
| Yes | 0.98 (0.94–1.02) | 0.39 | 1.00 (0.98–1.03) | 0.77 |
| **Four conditions as defined by the AOK Research Institute** |  |  |  |  |
| Arthrosis |  |  |  |  |
| No | Ref. | Ref. | Ref. | Ref. |
| Yes | 1.01 (0.99–1.02) | 0.46 | 1.05 (1.04–1.06) | <.001 |
| Chronic obstructive pulmonary disease |  |  |  |  |
| No | Ref. | Ref. | Ref. | Ref. |
| Yes | 1.12 (1.07–1.17) | <.001 | 1.16 (1.13–1.19) | <.001 |
| Asthma |  |  |  |  |
| No | Ref. | Ref. | Ref. | Ref. |
| Yes | 0.93 (0.90–0.97) | 0.001 | 0.92 (0.90–0.94) | <.001 |
| Cogenital heart disease |  |  |  |  |
| No | Ref. | Ref. | Ref. | Ref. |
| Yes | 0.96 (0.94–0.98) | <.001 | 1.00 (0.99–1.01) | 0.79 |
| **Typical geriatric conditions** |  |  |  |  |
| Decubitus |  |  |  |  |
| No | Ref. | Ref. | Ref. | Ref. |
| Yes | 1.28 (1.09–1.49) | 0.002 | 1.36 (1.23–1.49) | <.001 |
| Incontinence |  |  |  |  |
| No | Ref. | Ref. | Ref. | Ref. |
| Yes | 1.04 (0.96–1.13) | 0.29 | 1.04 (0.99–1.09) | 0.09 |
| Frailty |  |  |  |  |
| No | Ref. | Ref. | Ref. | Ref. |
| Yes | 1.22 (0.79–1.90) | 0.37 | 1.02 (0.75–1.38) | 0.92 |
| High risk of complications |  |  |  |  |
| No | Ref. | Ref. | Ref. | Ref. |
| Yes | 1.02 (1.00–1.05) | 0.09 | 1.02 (1.00–1.03) | 0.04 |
| Immobility |  |  |  |  |
| No | Ref. | Ref. | Ref. | Ref. |
| Yes | 1.16 (0.90–1.51) | 0.25 | 0.98 (0.83–1.15) | 0.80 |
| Cognitive deficits |  |  |  |  |
| No | Ref. | Ref. | Ref. | Ref. |
| Yes | 1.36 (1.25–1.47) | <.001 | 1.43 (1.36–1.51) | <.001 |
| Medication-associated problems |  |  |  |  |
| No | Ref. | Ref. | Ref. | Ref. |
| Yes | 0.91 (0.83–1.01) | 0.07 | 0.97 (0.92–1.02) | 0.26 |
| Pain |  |  |  |  |
| No | Ref. | Ref. | Ref. | Ref. |
| Yes | 1.02 (0.98–1.07) | 0.36 | 1.05 (1.02–1.08) | 0.001 |
| Sensibility disorders |  |  |  |  |
| No | Ref. | Ref. | Ref. | Ref. |
| Yes | 1.07 (1.00–1.14) | 0.04 | 1.08 (1.04–1.13) | <.001 |
| Loss of hearing and sight |  |  |  |  |
| No | Ref. | Ref. | Ref. | Ref. |
| Yes | 1.01 (0.96–1.06) | 0.84 | 1.02 (0.99–1.05) | 0.24 |
| Risk of falling and vertigo |  |  |  |  |
| No | Ref. | Ref. | Ref. | Ref. |
| Yes | 1.00 (0.95–1.04) | 0.89 | 1.02 (0.99–1.04) | 0.26 |
| Delayed convalescence |  |  |  |  |
| No | Ref. | Ref. | Ref. | Ref. |
| Yes | 0.77 (0.50–1.20) | 0.25 | 1.02 (0.79–1.32) | 0.86 |
|  |  |  |  |  |
|  |  |  |  |  |
